# Mapping of facial and vocal processing in common marmosets with ultra-high field fMRI

**DOI:** 10.1038/s42003-024-06002-1

**Published:** 2024-03-13

**Authors:** Audrey Dureux, Alessandro Zanini, Stefan Everling

**Affiliations:** 1 Centre for Functional and Metabolic Mapping, Robarts Research Institute, University of Western Ontario, London, ON N6A 5K8 Canada; 2 Department of Physiology and Pharmacology, University of Western Ontario, London, ON N6A 5K8 Canada

**Keywords:** Cognitive neuroscience, Social neuroscience

## Abstract

Primate communication relies on multimodal cues, such as vision and audition, to facilitate the exchange of intentions, enable social interactions, avoid predators, and foster group cohesion during daily activities. Understanding the integration of facial and vocal signals is pivotal to comprehend social interaction. In this study, we acquire whole-brain ultra-high field (9.4 T) fMRI data from awake marmosets (*Callithrix jacchus*) to explore brain responses to unimodal and combined facial and vocal stimuli. Our findings reveal that the multisensory condition not only intensifies activations in the occipito-temporal face patches and auditory voice patches but also engages a more extensive network that includes additional parietal, prefrontal and cingulate areas, compared to the summed responses of the unimodal conditions. By uncovering the neural network underlying multisensory audiovisual integration in marmosets, this study highlights the efficiency and adaptability of the marmoset brain in processing facial and vocal social signals, providing significant insights into primate social communication.

## Abstract (web summary)

9.4 T fMRI in marmosets shows a specialized brain network for face-voice cues processing, involving face patches and vocal areas along with parietal, prefrontal and cingulate cortices allowing complex social communication.

## Introduction

Primates emit a variety of signals during daily social communication, expressing specific emotional states, intentions, activities, or responses to external environmental features^1^. These complex signals encompass visual, tactile, olfactory and auditory cues^2^, with facial expressions and vocalizations serving as the primary sources for face-to-face primate communication, a notion already postulated by Charles Darwin^3^. The crossmodal integration of facial expressions and vocalizations is vital for perceiving a conspecific’s vocalization and concurrent facial behavior^4,5^.

Functional magnetic resonance imaging (fMRI) studies have revealed that humans, Old-World macaque monkeys, and New-World marmosets share a face processing system, consisting of interconnected patches distributed across the temporal and prefrontal cortex. In humans, face-selective patches are found in the lateral occipital cortex, the fusiform gyrus and in anterior and posterior regions of the superior temporal sulcus (STS)^6–8^. In macaques, these patches are located along the occipitotemporal axis mainly along the STS and in the frontal cortex^9–15^. In marmosets, similar patches have been identified along the occipitotemporal axis and in the lateral frontal cortex^16–18^, following a similar organization as in macaques and humans^11,19^.

Several studies have also associated the processing of negative facial expressions with higher activations in temporal face-selective regions and in prefrontal and subcortical areas in both humans and macaques^6,20–25^, a pattern that we also recently observed in marmosets^18^. Furthermore, vocalizations and vocal production contribute to interaction and cohesion within primate groups^26–28^. fMRI studies in humans have identified three voice-selective patches located along the mid-superior temporal gyrus to the anterior superior temporal gyrus (TVAa, TVAm, TVAp) and in premotor and inferior frontal areas^29–35^.

In macaques, two clusters in the STS have been identified with stronger activations for vocalizations than for other sounds categories^36–39^. Additionally, the recent discovery of a vocalization-selective cluster in the macaque anterior temporal pole suggests a similar functional organization of higher-level auditory cortex in macaques and humans^39^. These results suggest that vocalization processing is organized in ‘voice patches’ in the temporal lobe, analogous to the well-establish ‘face-patches’^36,40,41^.

Recently, we identified in marmosets a network akin to what was seen in humans. Vocalization-selective activations were observed in temporal, frontal and anterior cingulate cortices^42^. Furthermore, three voice patches were discerned along the STS, potentially homologous to the three human voice patches^42,43^.

The association between facial expressions and specific vocalizations enables a nuanced understanding of social cues, enhancing communication and social bonding among primate groups^5^. Yet, even though facial and vocal patches in primates have been individually studied, the process of integrating these multisensory signals during social interactions remains intricate and not fully understood.

Recent studies have begun to elucidate the neural substrates of multisensory integration in primates, demonstrating that audiovisual integration of social cues—specifically, faces and vocalizations—occurs in particular regions of the monkey face-patch and voice-patch systems^44–47^ as well as in the ventral lateral prefrontal cortex (VLPFC)^48–50^. Human studies have also highlighted the involvement of temporal^51,52^, frontal^53,54^, and parietal^53^ cortices in the processing of combined visual and auditory social information.

The field of multisensory integration in primate communication has been extensively explored in Old-World primates and humans. However, in New-World monkeys, the common marmoset (*Callithrix jacchus*) presents distinctive social behaviors, such as cooperative care of offspring and complex vocal communication, which are less common in Old-World species^4,55,56^. These unique characteristics of marmosets provide a valuable comparative model that can enhance our understanding of the evolution of neural mechanisms underlying social behaviors and inform the study of human social cognition and disorders^57–60^. In this context, the present study aims to explore the neural circuits for social multisensory integration in the common marmoset, an area so far unexplored. Utilizing ultra-high field MRI, we acquired whole-brain fMRI data from six awake marmosets while the animals were presented with videos of conspecific faces with no sounds, conspecific vocalizations with no videos, videos of conspecific faces with corresponding vocalizations, and scrambled versions of each of these conditions. By mapping these neural networks, we not only fill a significant gap in the literature but also provide insights that may be crucial for understanding the evolution of primate social communication and its implications for human social cognition, offering a potential avenue for translational research into social behavior and its disorders.

## Results

**Fig. 1:**
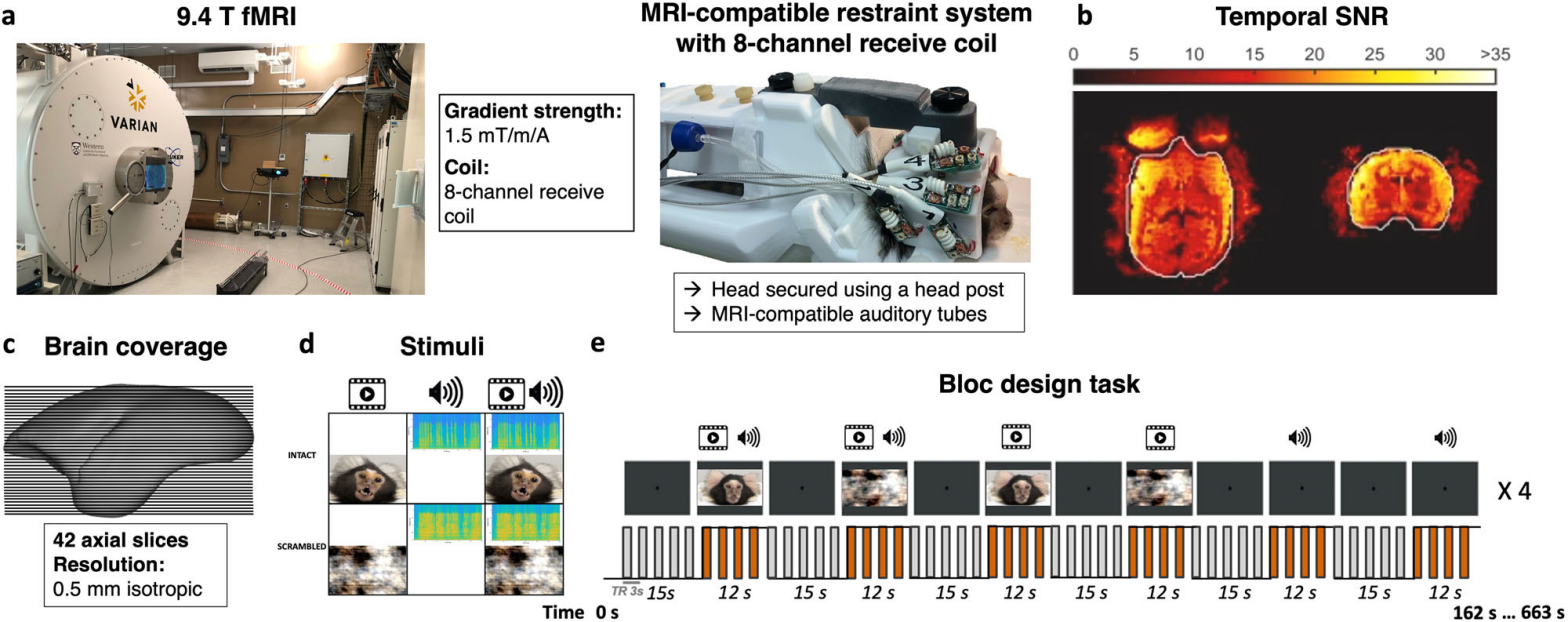
Overview of fMRI Methodology and Experimental Design. **a** Photograph of the 9.4 Tesla (T) ultra-high field MRI scanner used for functional imaging with details about specifications of the gradient strength and the multi-channel receive coil (left), and the custom MRI-compatible restraint system featuring an 8-channel receive coil, with a marmoset secured by a head post and equipped with MRI-compatible auditory tubes (right). All elements were photographed by the authors. **b** Representation of the temporal signal-to-noise ratio (tSNR) achieved at 9.4 T, indicating the high-quality data acquisition enabled (adapted from^61^ with permission). **c** Schematic of brain coverage, designed by the authors, illustrating the fMRI scan achieved through 42 axial slices with an isotropic resolution of 0.5 mm, superimposed on the NIH marmoset brain template^104^ (publicly available). **d** Depiction of the stimuli used in the experiment, categorized into three intact and three scrambled conditions: 1) unimodal videos of marmoset faces, 2) unimodal marmoset vocalizations, and 3) multimodal presentation of marmoset faces with corresponding vocalizations. Screenshots from the original recorded videos and custom-generated sound histograms, alongside custom-created icons for movies and sounds in PowerPoint, are by the authors. **e** Schematic of the sparse fMRI block design created by the authors, showing the temporal sequence of the presentation of intact and scrambled stimuli, each lasting 12 s, interspersed with 15 s baseline periods marked by a central fixation dot. During each run, the six conditions were presented in a randomized order and repeated four times, resulting in a total of 24 stimulus blocks and 25 baseline blocks. Each 3-second repetition time (TR) included a silent period of 1.5 s to ensure accurate perception of auditory stimuli.

In this study, we employed ultra-high field fMRI at 9.4 T to examine the neural correlates of multisensory processing in six awake common marmoset monkeys. Data were collected using a custom-built gradient coil with a 15 cm inner diameter and a maximum gradient strength of 1.5 mT/m/A, coupled with an 8-channel receive coil^61^ (Fig. 1a). The image quality of the functional runs was assessed by the temporal signal-to-noise ratio (tSNR), demonstrating high-quality data acquisition (Fig. 1b).

During scanning sessions, monkeys were positioned in a sphinx posture within an MRI-compatible restraint system, with their heads secured using a head post and MRI-compatible auditory tubes placed directly into their ear canals.

We acquired eight functional runs per animal, covering the whole brain with 42 axial slices at an isotropic resolution of 0.5 mm (Fig. 1c). To mitigate the masking of auditory stimuli by scanner noise, we implemented a continuous acquisition paradigm incorporating silent periods of 1.5 s within each 3-second TR^42^. Our block-design experiment utilized various stimuli, including marmoset face videos, vocalizations, marmoset face videos with corresponding vocalizations, and their scrambled versions, each presented for 12 s in a randomized sequence interspersed with 15-second baseline periods (Fig. 1d, e).

For detailed information on all methodological specifics, including surgical and anesthesia procedures, MRI training, positioning of marmosets within the MRI-compatible body restraint, fMRI scanning at 9.4 T, and preprocessing and statistical analysis of functional images, please refer to the methods section and the recent protocol paper by our group^62^.

Our primary objective was to uncover the neural architecture responsible for processing and integrating face and vocal signals in marmosets. Specifically, we sought to identify the brain regions responsive to face and vocalization processing, and the regions that were  activated by these combined signals. Our analysis included the creation of conjunction maps to explore both specific and common activations among these conditions, and the examination of the superadditive effect to investigate the responses to combined audiovisual stimulation.

### Functional brain activations during the processing of visual signals

**Fig. 2:**
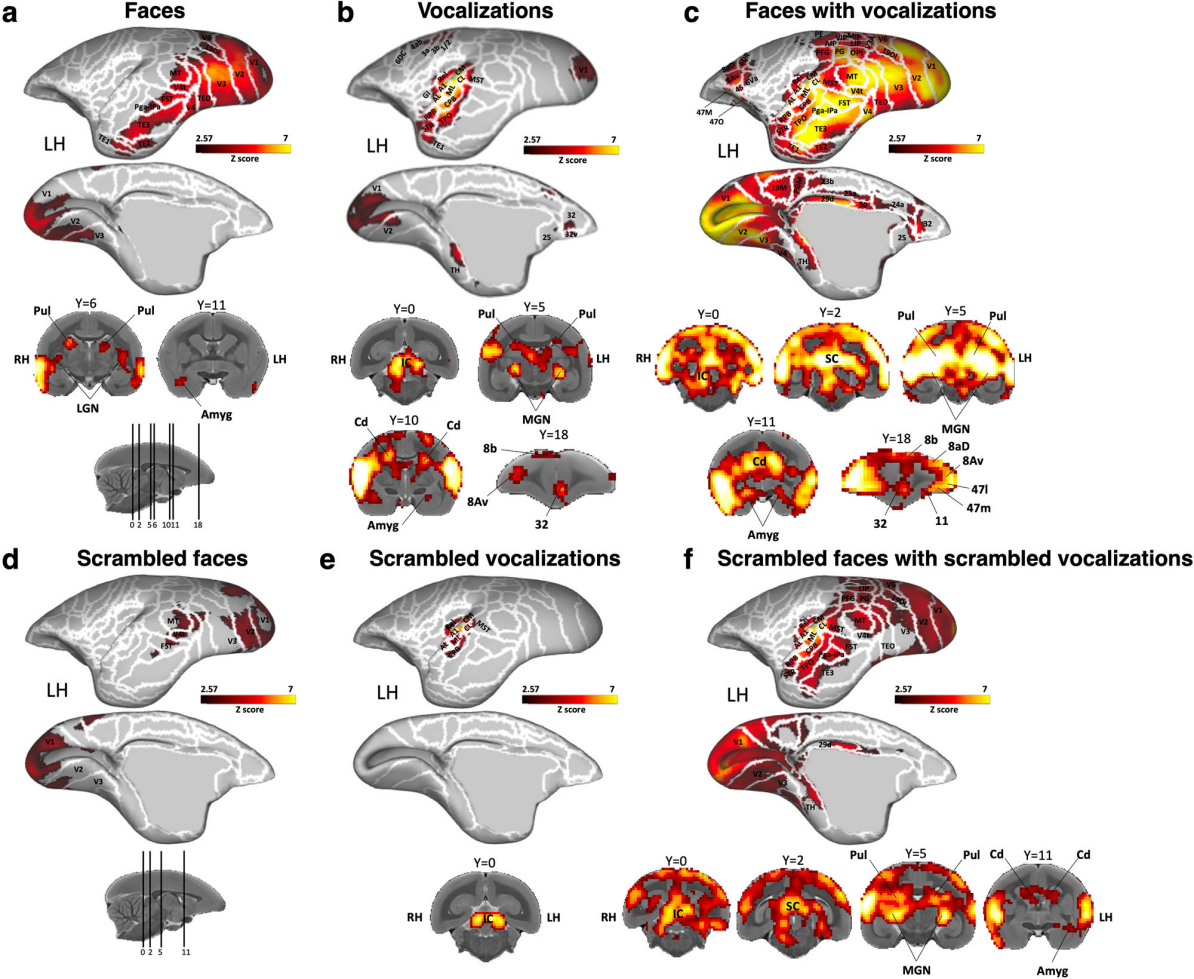
Brain networks activated by each condition *versus* baseline. This figure represents the group functional maps for each condition, showing significantly greater activations compared to baseline for marmoset face videos (**a**), marmoset vocalizations (**b**), marmoset face videos with corresponding vocalizations (**c**), scrambled marmoset face videos (**d**), scrambled marmoset vocalizations (**e**), and scrambled marmoset face videos with corresponding scrambled vocalizations (**f**). These group maps are based on data from six awake marmosets and are displayed on both lateral and medial views of the fiducial marmoset cortical surfaces, left hemisphere. Subcortical activations are represented on coronal slices. The white line delineates the regions based on the Paxinos parcellation^106^ of the NIH marmoset brain atlas^104^. The reported brain areas meet an activation threshold corresponding to z-scores > 2.57 (p < 0.01, AFNI’s 3dttest + +, cluster-size correction α = 0.05 from 10000 Monte-Carlo simulations).

Initially, we examined the processing of marmoset face videos and their corresponding scrambled versions, compared to a baseline period where only a central dot was presented on the screen. The group activation maps for each condition, focusing on the left hemisphere, are depicted in Fig. 2a, d. For a detailed visualization of the activation maps of the right hemisphere, see Supplementary Fig. 1a et 1d. Marmoset face videos (Fig. 2a) recruited a bilateral network primarily along the occipitotemporal axis, encompassing visual areas V1, V2, V3, V4, V4T, MT, V6, dorsointermediate part (19DI), lateral and inferior temporal areas TE1, TE2, TE3, TEO, the fundus of the superior temporal sulcus (FST), PGa-IPa, and ventral temporal areas 35, 36. The scrambled face videos (Fig. 2d) activated visual areas V1, V2, V3, V4T, MT, and the FST area. Subcortically, bilateral pulvinar, lateral geniculate nucleus (LGN) and right amygdala were recruited by face videos, whereas no activations were observed for scrambled faces.

**Fig. 3:**
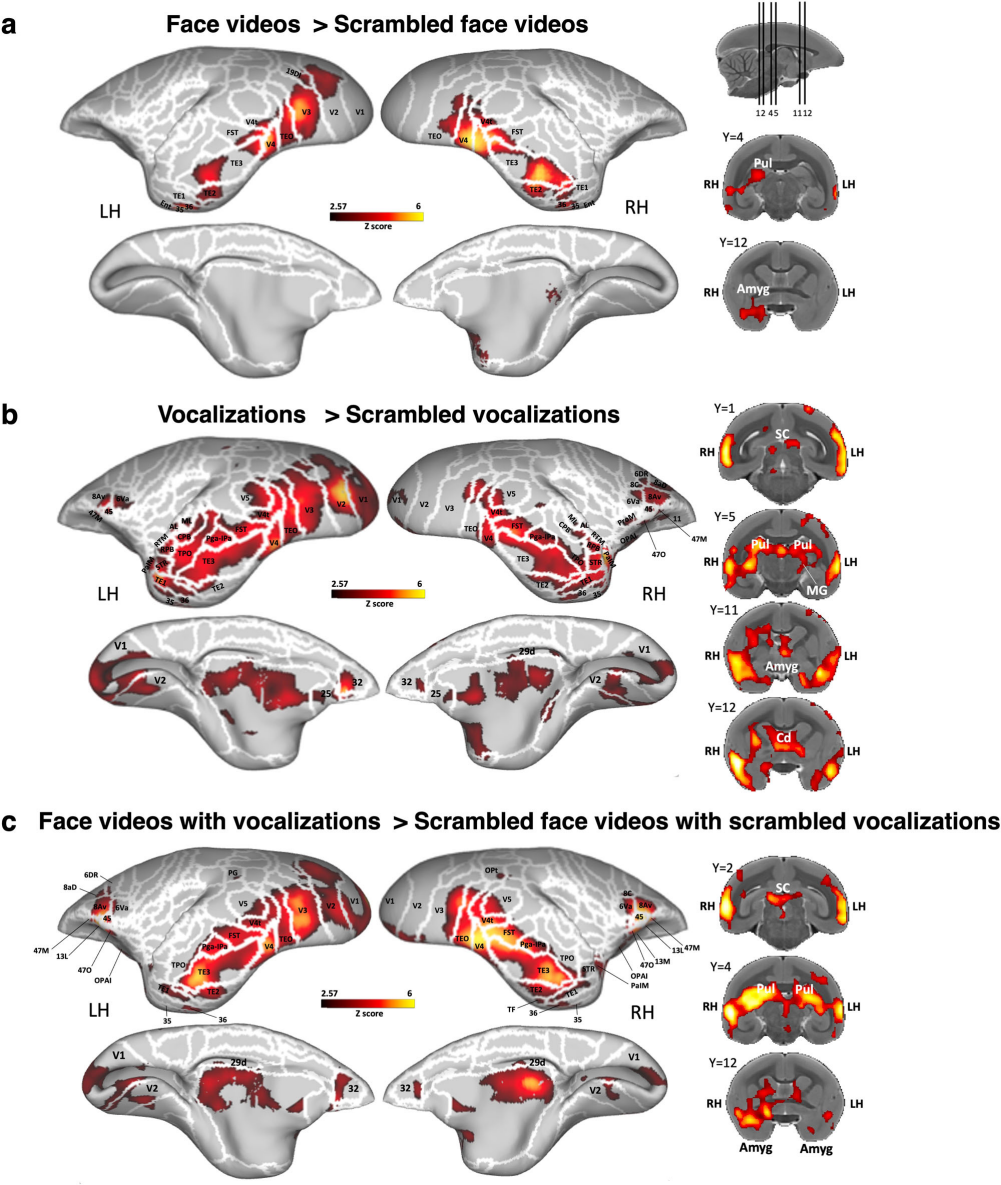
Brain networks involved in processing intact *versus* scrambled conditions. The group functional maps illustrate significantly greater activations for the comparison between (**a**) marmoset face videos and scrambled marmoset face videos, **b** marmoset vocalizations and scrambled marmoset vocalizations, and (**c**) marmoset face videos paired with corresponding vocalizations and their scrambled versions. These group functional topology comparisons are displayed on both the left and right fiducial marmoset cortical surfaces (lateral and medial views), as well as on coronal slices, to emphasize activations in subcortical areas. Regions are delineated by white lines, according to the Paxinos parcellation^106^ of the NIH marmoset brain atlas^104^. Reported brain areas have an activation threshold corresponding to z-scores > 2.57 (*p* < 0.01, AFNI’s 3dttest + +, cluster-size correction α = 0.05 from 10000 Monte-Carlo simulations).

Next, we identified brain regions more active during marmoset face observation by comparing the marmoset face videos condition with the scrambled condition (i.e., marmoset face videos > scrambled marmoset face videos contrast). The group activation map depicted in Fig. 3a revealed significant activations for marmoset faces compared to scrambled faces in a bilateral network comprising regions in the occipital cortex (i.e., V1, V2, V3, V4, V4t), and the temporal cortex (i.e., TEO, FST, TE1, TE2, TE3, 35, 36, entorhinal cortex). We also observed higher activations in area 19DI on the left hemisphere and in subcortical areas in the right pulvinar and the right amygdala (Fig. 3a).

For detailed quantitative comparisons of activation levels in various cortical regions under these conditions, refer to the bar graphs presented in Supplementary Fig. 2. These graphs visually demonstrate the variations in response levels across conditions and provide the statistical insights into the differences between them.

### Functional brain activations during the processing of auditory signals

Next, we explored the activation patterns for vocalization processing by analyzing each auditory condition – marmoset vocalizations and scrambled marmoset vocalizations – compared to the baseline period. The group activation maps for these conditions, focusing on the left hemisphere, are depicted in Fig. 2b, e. For detailed visualization of the activation maps of the right hemisphere under these conditions, see Supplementary Fig. 1b, e. For quantitative comparisons of activation levels in various cortical regions under these auditory conditions, refer to the bar graphs presented in Supplementary Fig. 2 and to the Supplementary Table 1.

Marmoset vocalizations (Fig. 2b) elicited bilateral brain activations in primary auditory cortex, including the core (primary area [A1] and rostral field [R], rostral temporal [RT]), belt (caudomedial [CM], caudolateral [CL], mediolateral [ML], rostromedial [RM], anterolateral [AL], rostrotemporal medial [RTM], rostrotemporal lateral [RTL]), and parabelt areas (caudal parabelt [CPB], rostral parabelt [RPB]). Additionally, activations were found in V1, V2, GI, TE1, TH, the medial superior temporal area (MST), the temporo-parietal-occipital area (TPO), the superior temporal rostral area (STR), the retroinsular area (ReI), as well as in bilateral frontal areas, including primary motor cortex 4ab, somatosensory cortex areas 3a, 3b and 1/2, premotor areas 6DR, 6DC, cingulate area 24c and in left cingulate areas 32, 32 v and 25. In the right hemisphere, there were also activations in S2I, DI, Ipro, agranular insular cortex (AI), medial part of parainsular cortex (PaIM) and 8aV.

Scrambled vocalizations (Fig. 2e) elicited responses mostly confined to the auditory cortex, with bilateral activations in core (A1, R, RT), belt (CM, CL, ML, RM, AL, RTM, RTL) and parabelt (CPB) cortices, as well as activations in the right hemisphere in adjacent areas TPO, MST, STR, GI, GI, DI, S2I, Ipro and PaIM.

To directly identify cortical and subcortical clusters that were more active for vocalizations, we compared the vocal to the scrambled conditions (i.e., marmoset vocalizations > scrambled marmoset vocalizations contrast). The group map in Fig. 3b shows stronger activations for vocalizations in R and RT areas of the core auditory cortex, in AL, ML, RTM, RTL areas of the belt auditory cortex and in RPB and CPM areas of the parabelt auditory cortex. We also found higher activations in occipito-temporal cortex in V1, V2, V2, V4, V4t, V5, TEO, FST, Pga-IPa, TPO, TE3, TE2, TE1, STR, 36, 35, Ent, and PaIM areas. More anteriorly, we found greater activations in the premotor cortex in bilateral area 6 ventral part (6Va) and in right areas 8 caudal part (8 C) and 6DR; in the frontal cortex in bilateral areas 8Av, 45 and 47 medial part (47 M) as well as in the right orbitofrontal cortex in areas 47 O, 13 lateral (13 L), 11 and orbital periallocortex (OPAI). Finally, higher activations were found in bilateral rostral cingulate areas 25, 32, and 29d. At the subcortical level, vocalizations induced stronger activations in the superior colliculus (SC), medial geniculate nucleus (MGN), caudate, pulvinar, and amygdala (Fig. 3b).

### Functional brain activations during the processing of audiovisual signals

In the intact audiovisual condition, as illustrated in Fig. 2c (see Supplementary Fig. 1c for visualization of the right hemisphere) - where marmoset faces were combined with corresponding vocalizations - we identified activations that reflected the combination of the previously described unimodal maps (Fig. 2a, b), alongside additional parietal, cingulate and frontal areas (Fig. 2c). Specifically, we observed activations in the previously mentioned areas along the occipital-temporal axis (i.e., bilateral V1, V2, V2, V4, V4t, MT, 19DI, TEO, MST, FST, Pga-IPa, TPO, TE3, TE2, TE1, STR, 36, 35, Ent), in auditory regions of the core, belt and parabelt cortices (i.e., bilateral A1, R, RT, CM, CL, ML, RM, AL, RTM, RTL, RPB, CPB), as well as in regions of the prefrontal and premotor cortices (i.e., bilateral 8aD, 8Av, 6Va, 45, 47 M, 47 O and 6DR). Beyond these areas, we found activations in bilateral posterior parietal areas surrounding the intraparietal sulcus (IPS), including the occipito-pareital transitional areas (OPt), and the anterior, lateral, medial and ventral intraparietal areas (AIP, LIP, MIP and VIP), as well as PG, PFG, PE and PGM areas. Within the cingulate cortex, activations were present in bilateral areas 32, 25, 24a, 23b, 30 and 29d.

In the scrambled audiovisual condition depicted in Fig. 2f (see Supplementary Fig. 1f for visualization of the right hemisphere) - where scrambled marmoset faces were associated with scrambled corresponding vocalizations - there were strong activations in bilateral auditory areas (i.e., A1, R, RT, CM, CL, ML, RM, AL, RTM, RTL, RPB, CPB), and in adjacent areas STR, TPO, MST, ReI, FST and Pga-IPa. Temporal areas TE3 and TEO, visual areas V1, V2, V3, V4t, MT, V6, as well as parietal areas LIP, PFG and PG were also recruited. In the right hemisphere, we also observed activations in areas 8Av, 45, and in the insular areas GI, DI, AI.

Quantitative comparisons of activation levels in these cortical regions are further detailed in Supplementary Fig. 2.

When comparing marmoset faces paired with corresponding vocalizations to their scrambled versions (Fig. 3c) (i.e., marmoset face videos with corresponding vocalizations condition > scrambled marmoset face videos with corresponding scrambled vocalizations condition), we found stronger activations for the intact stimuli in the occipitotemporal, frontal and orbitofrontal cortices in bilateral areas V1, V2, V3, V4, MT, V4t, FST, Pga-IPa, TPO, TE3, TE2, TE1, 35, 36, 8Av, 6Va, 45, 47 M, 13 L, 47 O, OPAI, as well as in left areas 8aD, 6DR, 13 M and in right areas 8 C and PaIM. However, no greater activations were found in the primary auditory cortex. Subcortically, greater activations were found in SC, pulvinar and amygdala (Fig. 3c).

Our results indicate that the integration of audiovisual signals involves a broad network, which includes not only the distinct face and vocal processing networks but also extends to encompass parietal, cingulate, and prefrontal regions. Notably, the intact and coherent pairing of marmoset faces with vocalizations—compared to their scrambled and incoherent counterparts—showed preferential processing along the occipitotemporal axis, in both the lateral and medial prefrontal cortices, and within the anterior cingulate cortex, especially area 32.

### Common and distinct brain regions involved in processing visual, auditory and audiovisual signals

**Fig. 4:**
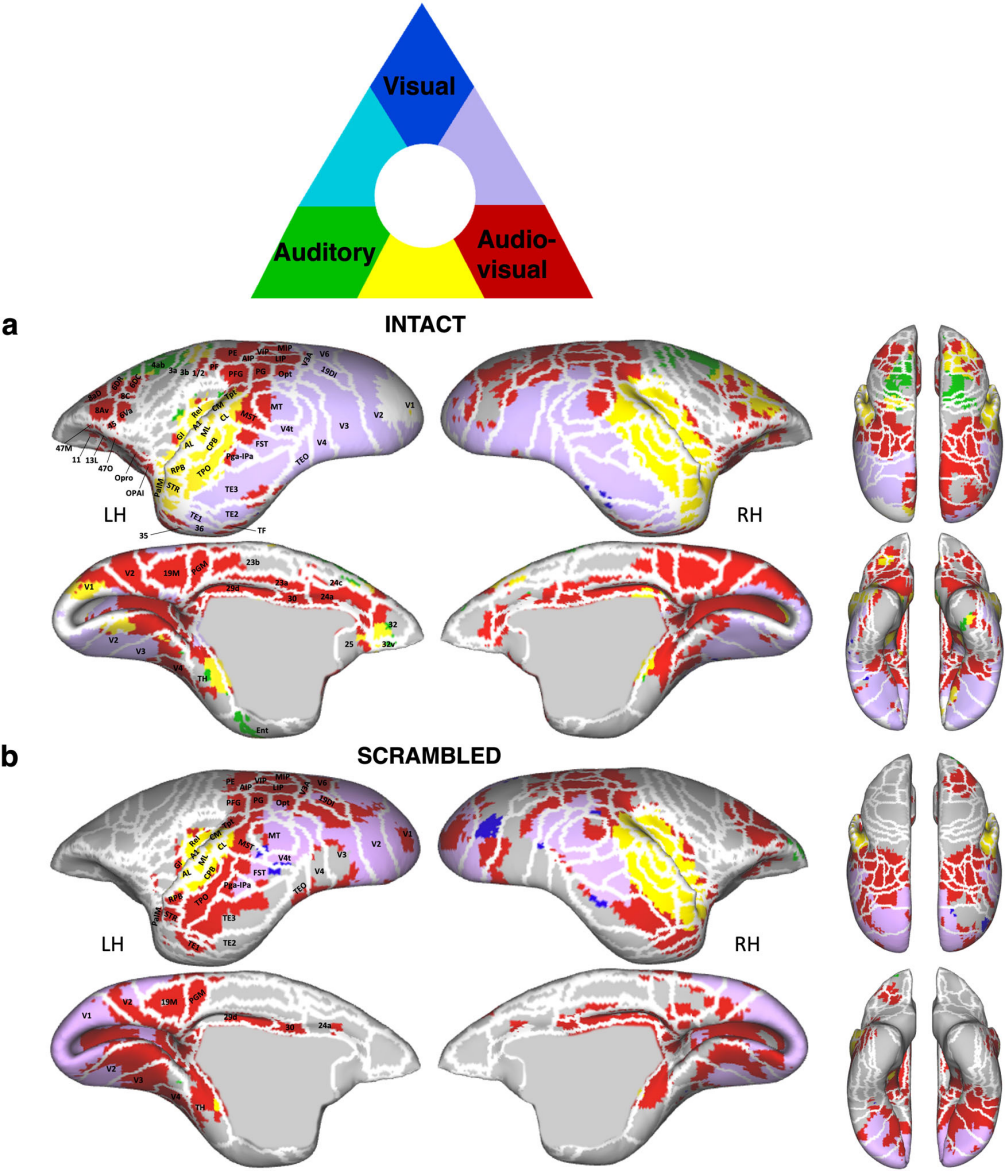
Spatial overlap of cluster networks for visual, auditory, and audiovisual processing in intact and scrambled conditions. Marmoset cortical surfaces for intact (**a**) and scrambled (**b**) conditions of both hemispheres are shown, displaying all significant voxels (z-scores > 2.57; *p* < 0.01, AFNI’s 3dttest + +, cluster-size correction α = 0.05 from 10000 Monte-Carlo simulations) as blue (unimodal visual condition > baseline), green (unimodal auditory condition > baseline), or red (audiovisual condition > baseline). Network overlap is indicated by the color key above the surface maps. Regions are delineated by white lines, according to the Paxinos parcellation^106^ of the NIH marmoset brain atlas^104^.

To identify common and distinct brain regions engaged in processing visual, auditory, and audiovisual modalities, we conducted a conjunction analysis separately for intact and scrambled conditions. The results of this analysis are displayed in Fig. 4a (intact stimuli) and Fig. 4b (scrambled stimuli). Our findings revealed a substantial overlap between the visual and auditory activation maps with the multisensory map (depicted in yellow and purple in Fig. 4).

Significantly, additional brain regions in the frontal, cingulate, and parietal cortices were found to be engaged only during multisensory stimulation (shown in red in Fig. 4). This observation suggests that multisensory processing is not a simple summation of each modality but also involves additional brain regions. Both the intact and scrambled maps selectively activated bilateral posterior parietal areas (i.e., AIP, LIP, MIP, VIP, PG, PFG, PE, PGM) and cingulate areas (i.e., 29d, 30, 24a), with the intact condition further recruiting other cingulate areas (23b, 23a, 24c) and portions of rostral cingulate areas 25 and 32. Interestingly, the anterior portion of area 32 was solely activated by auditory stimulation (shown in green in Fig. 4a), while its most posterior portion was only activated by multisensory stimulation (depicted in red in Fig. 4a). Between these, the region was commonly activated by both auditory and multisensory conditions (indicated in yellow in Fig. 4a).

In addition, certain prefrontal areas (8Av, 8aD) and premotor areas (6DR, 6DC) in the right hemisphere were shared between the auditory and multisensory intact maps (shown in yellow in Fig. 4a). However, other bilateral prefrontal (45, 47 M, 47 O) and orbitofrontal areas (11, 13 L, Opro, OPAI) were specifically activated by the intact multisensory condition (depicted in red in Fig. 4a).

For the scrambled condition, only portions of areas 6DR, 6DC, 8 C, 8Av, 8aD, 47 M, 47 O in the right hemisphere were activated by the audiovisual stimuli (Fig. 4b, shown in red).

Overall, for the intact audiovisual conditions, regions responding primarily to visual stimulation (and not auditory) were predominantly concentrated in the occipital and temporal cortices (Fig. 2a). In contrast, areas activated by auditory stimulation (but not visual) were situated in the primary auditory cortex, premotor cortex, and inferior frontal cortex (Fig. 2b). These regions also responded to combined audiovisual stimulation (Figs. 2c and 4a, shown in yellow and purple). Notably, no regions were identified that responded to both unimodal auditory and visual conditions (absence of light blue in Fig. 4a). Areas responsive to combined audiovisual stimulation, but not to unimodal visual and auditory stimulation, were distributed across frontal, cingulate, and parietal regions (Fig. 4a, shown in red). For the scrambled audiovisual conditions, regions responding to visual but not auditory stimuli remained concentrated in the occipital and temporal cortex, albeit to a lesser extent (Fig. 2d). The areas activated by auditory but not visual stimuli were primarily situated in the primary auditory cortex (Fig. 2e). These regions also responded to combined audiovisual stimulation (Figs. 2f and 4b, shown in yellow and purple). In this scrambled condition, areas solely responsive to audiovisual stimuli were mainly observed in parietal regions, with only a few in cingulate and right prefrontal regions (Fig. 4b, shown in red).

In summary, the intact, coherent conditions engage a more expansive neural network than the scrambled, incoherent ones, underscoring the intricate interplay of marmoset faces and vocalizations within the brain. The integration of these specific cues thus seems to engage extra brain regions, beyond those commonly activated by general audiovisual multisensory stimuli, as evidenced in the scrambled multimodal scenario.

**Fig. 5:**
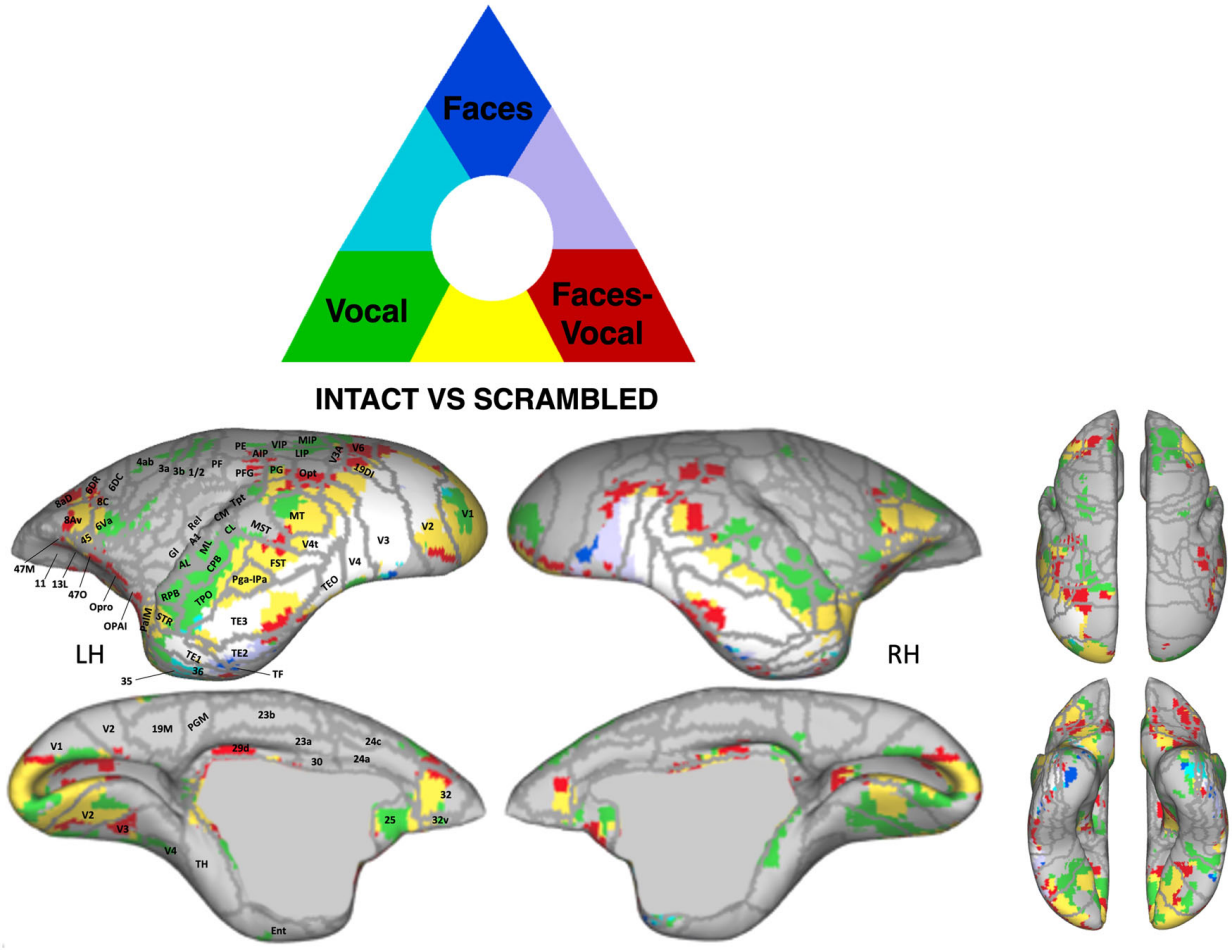
Spatial overlap of cluster networks for the comparison between intact and scrambled conditions for faces, vocalizations, and combined faces with corresponding vocalization. Marmoset cortical surfaces of both hemispheres are shown, displaying all significant voxels (z-scores > 2.57; *p* < 0.01, AFNI’s 3dttest + +, cluster-size correction α = 0.05 from 10000 Monte-Carlo simulations) as blue (unimodal condition marmoset faces > scrambled marmoset faces), green (unimodal condition marmoset vocalizations > marmoset scrambled vocalizations), or red (audiovisual condition marmoset faces with corresponding vocalizations > scrambled marmoset faces with corresponding scrambled vocalizations). Network overlap is indicated by the color key above the surface maps. Regions are delineated by white lines, according to the Paxinos parcellation^106^ of the NIH marmoset brain atlas^104^.

We subsequently conducted a conjunction analysis to compare intact versus scrambled stimuli for marmoset faces, vocalizations, and their combined presentation. This was done to discern the common and distinct brain regions involved in processing coherent social signals from faces, vocalizations, and their integrated form. As illustrated in Fig. 5, the green and blue regions demonstrate preferential activations for intact vocalizations and faces, respectively. The green regions encompassed the auditory cortex, including core, belt, and parabelt areas, and some motor and somatosensory areas, underscoring vocalization-specific processing. In contrast, the blue regions, localized to sections of temporal areas TE2 and TE1, signal face-specific responses. The red regions, predominantly located in the posterior parietal, prefrontal, and orbitofrontal cortices, highlight areas that are uniquely responsive to a coherent combination of marmoset faces and vocalizations, as opposed to their scrambled versions.

The overlaid regions, delineated in purple, clear blue, yellow, and white, signify converging neural responses between the different contrasts. Specifically, the white regions along the occipitotemporal axis, encompassing V2, V3, V4, V4t, TEO, and parts of FST, TE3, TE2, and TE1, denote a common response to all three intact versus scrambled stimulus categories.

The yellow regions, involving portions of prefrontal area 45, premotor area 6Va, FST, Pga-IPA, MT, STR, PaIM, and early visual areas V1 and V2, show a shared neural response for processing both vocalizations and the integration of faces with corresponding vocalizations. In line with our previous results (Fig. 4a), the anterior section of area 32 shows a distinct response to intact vocalizations (in green), the posterior section to the integrated face-vocalization stimuli (in red), and an intermediate zone to both (in yellow). Notably, no regions showed a dual response to both marmoset faces and vocalizations or to marmoset faces and the combination of faces and vocalizations (indicated by the absence of light blue and purple).

In summary, our findings from the conjunction analysis reveal a nuanced neural representation of social stimuli, distinguishing specific and shared processing networks for marmoset faces, vocalizations, and their integrated combinations. A consistent network across the occipitotemporal axis responds to all three modalities when comparing intact to scrambled versions, suggesting an ability to process complex social cues. This network, with the engagement of additional parietal and frontal regions, which are specifically recruited for processing the integrated presentation of marmoset faces with vocalizations, allow multisensory integration in the marmoset brain.

### Positive interaction for combined audiovisual signals: superadditive effect

To determine the superadditive effect – corresponding to an increased activation when subjects integrate multimodal information compared to the sum of activations from single-modality inputs - we created activation maps displaying regions with stronger activations for the combined auditory and visual face-related information conditions compared to the sum of responses from unimodal face and vocal conditions (i.e., face videos with corresponding vocalizations > face videos + vocalizations).

**Fig. 6:**
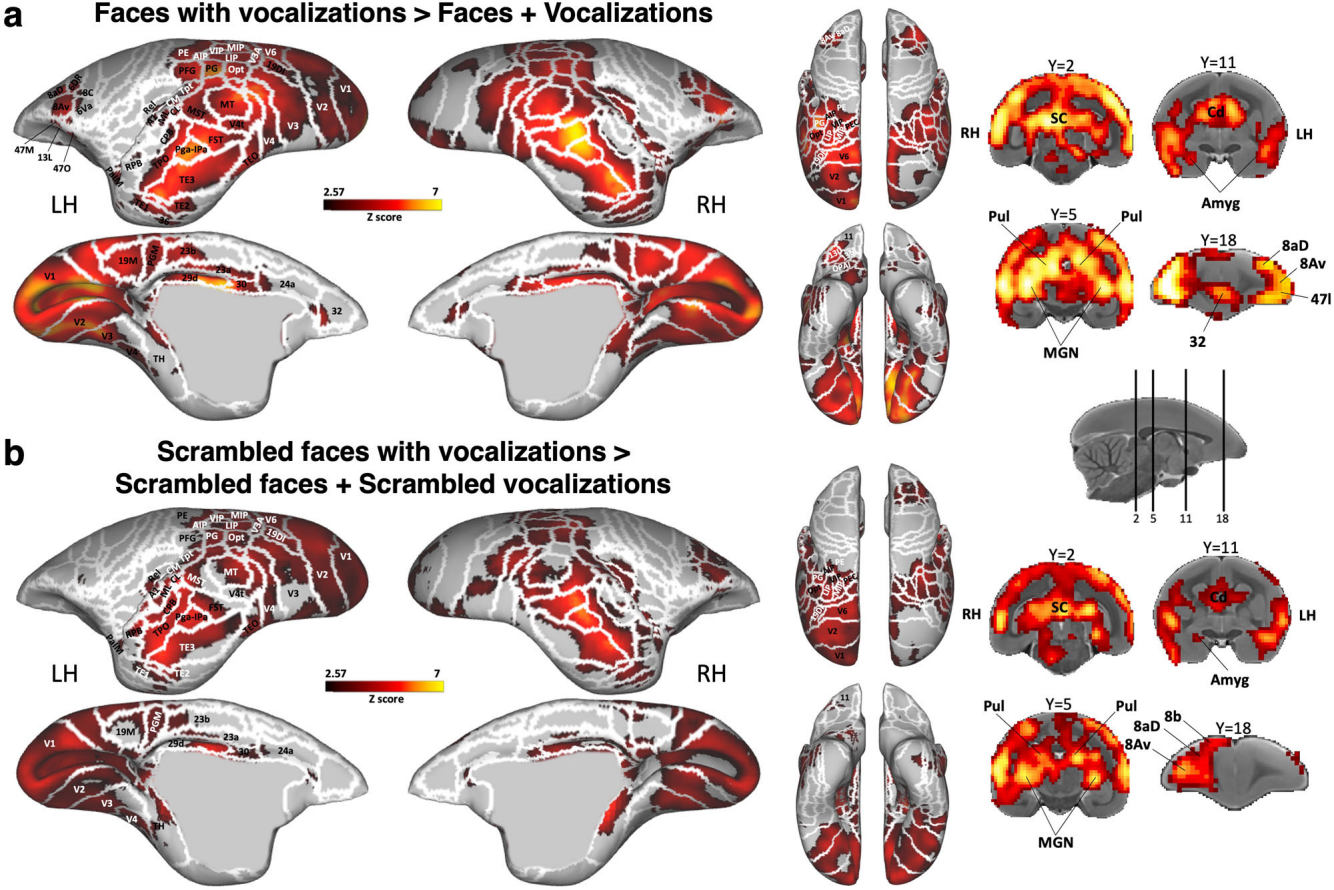
Superadditive neural processing of multisensory face and vocal signals. Group functional maps illustrate significantly greater responses to the multisensory audiovisual conditions compared to the sum of the responses for its unimodal constituents for both intact (**a**) and scrambled (**b**) stimuli. Significant differences were determined with paired t-tests, thresholded at z > 2.57 (*p* < 0.01, AFNI’s 3dttest + +, cluster-size correction α = 0.05 from 10000 Monte-Carlo simulations). The group functional topology comparisons are displayed on both left and right fiducial marmoset cortical surfaces, as well as on coronal slices. Regions are delineated by white lines, according to the Paxinos parcellation^106^ of the NIH marmoset brain atlas^104^.

This contrast, as illustrated in Fig. 6a for intact conditions and Fig. 6b for scrambled conditions, revealed that areas in the temporal (i.e., bilateral areas TEO, FST, MST, PGa-IPa, TE3, TE2, TE1, TPO, 36), parietal (i.e., bilateral areas MIP, LIP, VIP, AIP, PG, PE, PFG, PGM), cingulate (i.e., bilateral areas 23b, 23a, 29d, 30, 24a), as well as premotor and prefrontal (i.e., areas 8Av, 6va, 8 C, 8aD, 6DR, 47 M, 47 O bilateral for intact and right for scrambled) cortices responded more robustly to combined audiovisual conditions compared to the cumulative response of isolated auditory and visual conditions. Furthermore, while visual (i.e., bilateral areas V1, V2, V3, V4, MT, V6, V3A, V4t, 19DI) and auditory (comprising bilateral regions like A1, CM, ML, CL, CPB, RBP) cortices also displayed notable response differences between multimodal and unimodal stimulations, the most significant variance in activations was primarily located in the temporal, parietal, and cingulate regions.

Subcortically, structures such as the superior colliculus, caudate, MGN, pulvinar, and amygdala exhibited a stronger response to the multisensory condition compared to the combined responses of unimodal conditions. Overall, this multimodal enhancement effect was more pronounced and extensive for intact conditions compared to scrambled conditions. Notably, area 32 was activated solely by the intact contrast.

To provide a more detailed understanding of these observations, we included bar graphs in the supplementary information (Supplementary Fig. 2), which display the beta values of various regions of interest (ROIs) under the different conditions. Additionally, our statistical analysis, which compared the beta values in the multisensory condition (faces with vocalizations) against the combined beta values from the unimodal conditions (faces + vocalizations) across these ROIs, revealed significant superadditive effects in all the ROIs, except for the frontal region 11 of the left hemisphere (all *p*-values < 0.01, except for area 11 left hemisphere). This analysis underscores the nuanced and robust nature of the superadditive response, especially in regions involved in sensory integration and higher-order processing.

## Discussion

Our study aimed to identify the distinct and shared neural substrates responsible for processing marmoset faces, vocalizations, and the combination of marmoset faces with their associated vocalizations. Within this multisensory framework, we further examined how these integrated audiovisual signals are processed in comparison to unimodal auditory and visual stimuli. The ability to recognize and integrate social cues is crucial for effective communication. Our central hypothesis proposed that marmosets process face and vocal signals in distinct face and vocal patches within their temporal and frontal cortices, and that these patches should also be involved in audiovisual multisensory processing, with responses to combined stimuli exceeding the summed responses of individual auditory and visual stimuli - a phenomenon known as superadditivity.

To test this hypothesis, we utilized ultra-high field fMRI acquisitions and exposed awake marmosets to various stimuli, including marmoset face videos, vocalizations, and their corresponding scrambled versions both separately and in combination.

The ability to recognize faces is paramount in deciphering the intentions of others, making the differentiation and interpretation of facial expressions vital for social communication^63–65^. Previous fMRI research on face processing in human and nonhuman primates has identified face patches located across temporal and prefrontal cortices, which responded strongly to faces compared to non-face objects or scrambled faces^6–8,10,11,16–19^. Our results from visual stimulation depicting marmoset face videos, as well as comparisons of these videos to their scrambled versions, are consistent with recent investigations into face processing in marmosets. These results emphasize the role of occipito-temporal regions in processing faces and facial expressions^16–18^. These areas display robust activation in V2/V3, V4/TEO, V4t/FST, TE2-TE3, corresponding to the previously identified face-patches in marmosets (i.e., patches O (occipital), PV (posterior ventral), PD (posterior dorsal), MD (middle dorsal) and AD (anterior dorsal) respectively)^16,18^. Subcortically, face videos preferentially recruited the pulvinar, amygdala, and LGN, in contrast to scrambled faces which did not elicit the same responses. The selective activation of the LGN by face videos, and not by scrambled faces, may be influenced by cortical feedback mechanisms. The LGN is typically seen as a primary visual relay, but it is also subject to extensive feedback from cortical areas. This top-down influence from higher-order visual areas to the LGN is known to modulate sensory processing, potentially enhancing or suppressing neural responses based on the context and recognizability of stimuli^66,67^. In the case of our study, the coherent, structured nature of face videos could engage these feedback pathways more robustly compared to scrambled faces, which lack meaningful visual content. Such dynamics align with the concept that perception is not a mere bottom-up process but a complex interplay of sensory and cognitive factors^68^. Further research into these feedback mechanisms could elucidate how the brain discerns and prioritizes meaningful visual stimuli over less coherent ones.

Research in primate vocalization processing, another fundamental mode of communication in primates, has revealed the existence of selective voice patches in the temporal cortex, specifically along the STS, and in certain regions of the frontal cortex in humans, macaques, and marmosets^29–32,36–39,42,69^. Our results align with these findings and our recent investigation in marmosets^42^, showing similar activations in the primary auditory cortex, rostral cingulate, frontal, and temporal cortices in response to auditory stimulation playing vocalizations. Moreover, we observed comparable activations in the primary auditory cortex and MST for scrambled vocalizations. Intriguingly, our results show that activations in the MST region, for both vocalizations and scrambled vocalizations, are predominantly localized in its rostral part. This specific localization supports the hypothesis proposed by Majka et al.^70^, suggesting that this part of MST might functionally correspond to the caudal subdivision of the superior temporal polysensory area (TPOc), indicating a complex functional anatomy within MST for auditory processing.

Our analysis comparing vocalizations with their scrambled vocalizations has revealed additional activations in regions not observed in our initial study^42^, including areas in occipital, inferior and lateral temporal, as well as prefrontal cortices. This pattern could be attributed to a notable decrease in activity within the occipitotemporal regions during both conditions, with a more significant deactivation during the presentation of the scrambled conditions (refer to Supplementary Fig. 3 for visualization). Such deactivation was absent in our earlier auditory-only study^42^, suggesting that our current experimental design—incorporating visual and audiovisual stimuli either preceding or following the vocalization condition—may influence the neural processing of auditory signals.

Multisensory integration is defined as a process whereby the neuronal responses to two sensory inputs is different from the sum of the neuronal responses to each on its own^71,72^. Numerous fMRI studies in humans and several electrophysiological studies in macaques have demonstrated audiovisual multisensory interactions in the temporal cortex. Specifically, the STS has been identified as displaying multisensory responses in both humans and macaques, exhibiting enhanced activity for bimodal auditory and visual signals over unimodal ones. Human fMRI studies have confirmed the STS as a multisensory region^51,52^, emphasizing its specialization in integrating various types of information within modalities (e.g., visual form, visual motion) and across modalities (auditory and visual). In macaques, electrophysiological evidence has revealed that individual neurons in the STS may respond solely to auditory stimuli, exclusively to visual stimuli, or to both auditory and visual stimuli^73,74^. Additionally, some research has indicated that STS also harbors cells with selective audiovisual responses to faces^46,75,76^.

Beyond the STS, recent evidence of audiovisual integration during naturalistic social stimuli has been found in specific regions of the monkey face-patch system^47^, the voice-patch system^44–46^, and the prefrontal cortex with the presence of multisensory neurons in the ventrolateral prefrontal cortex (VLPFC) responsive to combined face and voice stimuli^48–50^.

Understanding the bimodal integration of visual and auditory signals in primates has previously been derived exclusively from human and macaque studies, leaving the processing of associations between vocalizations and dynamic faces in marmosets unexplored until now.

In our study, the multisensory condition, which combined marmoset face videos and corresponding vocalizations, increased the activity of face and voice patches, illustrating the similarity of these areas’ role in audiovisual multisensory integration in marmosets with what has been observed in macaques. Moreover, the activations seen in the TE complex (i.e., TE3, TE2, and TE1) could correspond to the STS responses observed in macaques and humans^19^, further showcasing multisensory integration in these regions in marmosets. A recent electrophysiology study in macaques established that neurons in the anterior fundus face patch in the STS (patch AF) responded to both visual and vocal stimuli, signifying their role in audiovisual integration during social communication^47^. Our findings support this, showing activations in the temporal face-patches regions during the integration of faces and vocalizations. The activations observed in the anterior face patches MD and AD might be equivalent to the AF patch in macaques^11^.

In the frontal cortex, we also observed activations in various prefrontal areas, unveiling their multisensory role during processing of simultaneous facial expressions and vocalizations. Significantly, the rostral cingulate area 32, previously found to be activated in response to vocalizations in marmosets^42^ and during language processing in humans^31,77,78^, was also engaged in voice-face multisensory processing in our study.

Our conjunction analysis revealed that the processing of audiovisual stimulation extended beyond the recruitment of areas solely responsible for unimodal visual and auditory processing. This included not only the regions that process face and vocal signals but also additional cortical areas, suggesting a more intricate network engaged in multisensory integration. This finding highlights the complexity of multisensory processing, shedding new light on the underlying neural mechanisms. The areas activated solely by the multisensory condition were primarily situated in parietal, frontal and cingulate cortices, indicating that these regions may also serve as integrative hubs necessary to process the integration of both visual and auditory information for more efficient social communication.

Firstly, our findings reveal a noteworthy pattern of bilateral posterior parietal activations in response to combined audiovisual stimuli, around the IPS and in parietal areas PF, PE, PFG and PGM. This finding aligns with and extends existing research conducted on humans and macaques, which implicates parietal areas in multisensory processing^79,80^. In humans, numerous fMRI studies have revealed the involvement of the IPS in tasks that demand the integration of visual and auditory stimuli^53^, aligning well with our present observations in marmosets. In macaques, although some studies suggest audiovisual integration in the posterior parietal cortex, responses to stimuli in bimodal conditions have not been directly examined^81^. Our results with marmosets parallel the findings observed in humans, underscoring the potential evolutionary conservation of this region’s role in multisensory processing across primates.

Secondly, our results reveal several areas in the frontal cortex situated in some lateral prefrontal (i.e., 47 M, 47 O, 45) and orbitofrontal areas (i.e., 11, 13 L, OPro, OPAI), as well as in posterior and anterior cingulate areas (i.e., 29d, 23d, 23a, 30 24a) responding only to the multisensory audiovisual condition. In macaques, the integration of auditory and visual information has been described in the VLPFC. Some studies have shown that single neurons in VLPFC integrate audiovisual species-species face and vocal communication stimuli, suggesting that these neurons are an essential node in the cortical network composed by unimodal auditory and visual regions responsible for communication^48–50^.

In humans, fMRI studies have also demonstrated the activation of inferior frontal gyrus during the processing and integration of speech and gestures^82,83^, suggesting a larger role of the IFG in communication than classical auditory-speech processing^84^. Some studies have further demonstrated a decrease activity in ventral prefrontal cortex for incongruent faces and voices^72,83^. In macaque monkey, the evidence shows that cells in the ventral PFC respond to and integrate audiovisual information, with some cells exhibiting multisensory enhancement or suppression when face-vocalization stimuli are combined^48,49^. In humans, the integration of audiovisual stimuli also occurred at the level of the anterior cingulate/medial prefrontal cortex^54^. It has been shown that the activity in these areas was enhanced during pairing of congruent and incongruent cross-modal visual and auditory stimuli, and the activation was greater during matching conditions^54^. These results align with our findings in marmosets, with activations for the combined audiovisual conditions in cingulate/medial prefrontal cortex. Thus, our results have allowed us to add a piece of evidence that the ventral frontal lobe and the cingulate cortex of primates may be involved in processing the association between a face or facial gesture and a vocal stimulus. This suggest that the PFC may be a precursor to the more complex functions of the human frontal lobe, where semantic meaning is linked with acoustic or visual symbols^49^.

Moreover, we observed specific activations in parts of the premotor regions (e.g., 6DC) and the primary motor cortex in response to intact marmoset vocalizations. These activations may reflect a preparatory response for orienting toward a vocalizing conspecific, which is essential for primate social interactions. This aligns with findings from Roy et al.^85^, who demonstrated the premotor cortex’s involvement both before and during self-initiated vocalizations when marmosets engaged in vocal exchanges with conspecifics. Notably, a subset of premotor cortex neurons was activated specifically by vocal production and not by other orofacial movements, such as licking. This suggests that the premotor cortex may either control specific muscles involved in vocal production, such as those in the larynx, or represent upstream control signals for initiating vocalization^85^. Our fMRI findings suggest an anticipatory neural mechanism in marmosets, potentially oriented towards the source of vocalizing conspecifics. Such a mechanism could be crucial for appropriate social responses in a context-dependent manner.

It is important to note that, although not previously reported in macaques and humans, we show that the rostral cingulate area 32 participates in the integration of visual and auditory stimuli. Interestingly, only the posterior portion of this area responded exclusively to multisensory stimulation. In contrast, the anterior portion was activated solely in response to auditory cues. The intervening region displayed activation in both auditory and combined audiovisual conditions. Nevertheless, these findings held true only for intact, coherent stimuli and not for the scrambled, incoherent versions. Reser et al.^86^ provide an important anatomical context to these findings. They observed that the marmoset RT area, part of the core auditory network, has direct projections to a region considered part of area 32. This anatomical connection might contribute to the role of area 32 in auditory cognition, as seen in our study. This underscores the complexity of the involvement of this area in processing auditory and audiovisual social cues.

While similarities exist in the neural networks processing multisensory stimuli between intact and scrambled conditions, the activations elicited by intact audiovisual stimuli were more pronounced and extensive, particularly in the prefrontal cortex, compared to their scrambled counterparts. This difference was less marked in the parietal cortex. Directly comparing the two conditions, we observed that intact audiovisual stimuli elicited greater activations across the occipito-temporal axis, within the prefrontal cortex, and notably in the rostral cingulate area 32. Subcortically, the SC, pulvinar and amygdala were also more activated in response to intact audiovisual condition. The distinct activation patterns within area 32, particularly the differential responses between its posterior and anterior portions, suggest a specialized role in integrating coherent and congruent multisensory information. Specifically, the posterior portion of area 32, which exclusively responds to multisensory stimulation, appears to play a key role in processing complex sensory information, aiding in distinguishing meaningful audiovisual social interactions from nonsensical or mismatched stimuli. This aligns with findings in humans where the anterior ACC is implicated in processing context-dependent multimodal events^87,88^. For instance, Laurienti et al.^54^ observed increased activity in the anterior cingulate gyrus and adjacent medial prefrontal cortex when auditory and visual stimuli were contextually congruent, as opposed to when they were mismatched. These observations highlight the advanced cognitive function of area 32 in marmosets, extending beyond basic sensory integration to encompass context and relevance in social communication. Such evidence suggests that the rostral anterior cingulate area 32 might serve as an integration center for context-dependent audiovisual information in social cognition, significantly influencing social interactions.

Furthermore, the differential activation observed across temporal, prefrontal, and cingulate regions in response to intact versus scrambled conditions could reflect a sophisticated neural mechanism. This mechanism is capable of differentiating relevant audiovisual social information from nonsensical or mismatched stimuli, thereby enhancing the precision and efficacy of primate social communication.

The principle of superadditivity, in which multisensory responses exceed the sum of the linear additive responses to the unimodal stimuli, has been advocated by some researchers as a requirement for brain regions involved in multisensory integration^89,90^. In line with this, we assessed the augmented responses to the audiovisual multimodal conditions in comparison to the sum of visual and auditory unimodal conditions, aiming to identify the regions exhibiting a superadditive effect. Our findings revealed that this augmented response was not merely a simple summation of unimodal stimuli, but rather a complex interplay of activation across temporal, parietal, cingulate, lateral, and medial prefrontal areas. This suggests a synergistic enhancement of sensory processing that goes beyond mere additive effects. Both visual and auditory areas also demonstrated significant differences between multimodal and unimodal stimulations, although the more pronounced differences in activations were situated in the temporal, parietal, and cingulate areas. Subcortically, structures such as the SC , caudate, MGN, pulvinar, and amygdala exhibited a stronger response to the multisensory condition compared to the sum of unimodal conditions. The superadditive effect, especially pronounced in these areas, contributes to the efficiency and robustness of multisensory integration, enhancing the marmosets’ ability to perceive, interpret, and respond to complex social cues.

These enhancements, transcending traditional models of sensory integration, resonate with the dynamic and context-sensitive neural mechanisms proposed by Ghazanfar and Schroeder^91^ and Stein and Stanford^92^. This intricate interplay between sensory modalities, particularly evident in social contexts, suggests a nuanced and perhaps evolutionarily conserved mechanism where the brain integrates complex social cues in a way that significantly enhances overall perceptual experience. This implies that the integration of social sensory information in marmosets, and potentially other primates, relies on sophisticated neural networks extending beyond mere sensory combination, playing a crucial role in the rapid and effective processing of social cues during communication^91,92^.

While our study focuses on the integration of audiovisual social cues, it is important to note that other forms of audiovisual integration likely involve different neural substrates. A recent meta-analysis summarizing 121 neuroimaging studies that examined the neural basis of audiovisual integration in humans, has demonstrated that the experimental context and stimulus complexity influence the brain networks identified during audiovisual integration^93^. Their results suggest that audiovisual integration can occur via engaging a network of different brain regions at multiple levels which are highly context-dependent. These include sensory sites (i.e., middle and inferior occipital gyrus, fusiform gyrus, lingual gyrus, and the middle portion of superior temporal gyrus), subcortical sites (i.e., thalamus), and higher association sites (i.e., superior temporal cortex and middle and superior frontal gyrus). These studies indicate that the neural pathways for audiovisual integration appear to be flexible rather than a fixed network of brain regions, with superior temporal cortex playing a central role in these neural assemblies. Furthermore, in macaques and marmosets, audiovisual integration for object/sound source localization has been shown to rely on distinct structures. Notably, the caudal subdivisions of auditory cortex and adjacent superior temporal polysensory areas have direct projections to the primary visual cortex. This architecture is hypothesized to enhance stimulus localization in peripheral spaces^70,94^. Such distinctions underline the complexity and specificity of multisensory processing across different contexts. Our study, by detailing the neural correlates of social cues integration, not only enriches the understanding of multisensory processing but also opens avenues for exploring how diverse forms of audiovisual integration come together to influence our overall sensory perception.

Furthermore, it is pertinent to address the nature of the stimuli used in our study, specifically the inclusion of negative facial expressions. Previous literature indicates that negative expressions tend to elicit more robust neural responses compared to neutral expressions in temporal, prefrontal, and subcortical areas in both humans and macaques, a pattern also observed in marmosets^6,18,20–25^. Therefore, our findings, which focused on negative expressions, may not fully represent the neural processing associated with other types of facial expressions. Future studies exploring a broader range of emotional expressions in marmosets are necessary to gain a comprehensive understanding of emotional processing in a multisensory social context in this species.

In summary, our findings reveal a conserved audiovisual multisensory pathway in marmosets, characterized by the activation of face patches along the occipitotemporal axis and vocal patches in the auditory cortex, intricately linked with prefrontal and cingulate areas. These results suggests that during audiovisual social communication, marmosets recruit a more extensive neural network than what is engaged during the processing of visual and auditory signals alone, highlighting the evolutionary importance of integrating facial and vocal cues for interpreting social interactions^4,55–57,95,96^. Given the marmosets’ rich social behaviors, which parallel human traits such as prosocial behavior, imitation, and cooperative breeding, our study offers a valuable perspective on the evolution of complex social communication processes within primates. It addresses a critical gap in our comparative understanding of primate neurobiology, illuminating both the specialized multisensory processing in this species and the potential evolutionary mechanisms shared across primates in social communication. Therefore, these results not only enhance our knowledge of marmoset neurobiology but also underscore the species’ potential as a model for translational research. This is particularly pertinent in the study of neurodevelopmental and psychiatric conditions affecting social interaction, where marmosets may provide key insights^60,97,98^.

## Methods

### Common Marmoset subjects

All experimental procedures were in accordance with the guidelines of the Canadian Council of Animal Care policy and a protocol approved by the Animal Care Committee of the University of Western Ontario Council on Animal Care #2021-111. We have complied with all relevant ethical regulations for animal use. Ultra-high field fMRI data were collected from six awake common marmoset monkeys (*Callithrix jacchus*): two females (weight 315 and 150 g, age 44 months) and four males (weight 365–459 g, age 32–44 months).

To prevent head motion during MRI acquisition, animals were surgically implanted with an MR-compatible machined PEEK (polyetheretherketone) head post^61^, conducted under anesthesia and aseptic conditions. During the surgical procedure (for details, see refs. ^62,99^, the animals were first sedated and intubated to ensure they remained under gas anesthesia, maintained by a mixture of O_2_ and isoflurane (0.5–3%). With their heads immobilized in a stereotactic apparatus, a machined PEEK head fixation post was positioned on the skull following a midline skin incision along the skull. This device was secured in place using a resin composite (Core-Flo DC Lite; Bisco). Heart rate, oxygen saturation, and body temperature were continuously monitored throughout the surgery. Two weeks post-surgery, the monkeys were acclimated to the head-fixation system and the MRI environment through a three-week training period in a mock scanner, as described in Gilbert et al.^100^.

### Multisensory facial expressions task and experimental setup

We utilized six different types of stimuli derived from previously recorded videos^18^. These videos originally depicted negative facial expressions. From these videos, we generated two video conditions, two audio conditions and two conditions involving both video and corresponding audio (Fig. 1d). We used custom video-editing software (iMovie, Apple Incorporated, CA) for this purpose. The two video conditions encompassed marmoset face videos with no sound and their scrambled versions. The two audio conditions consisted of vocalizations extracted from the videos and their scrambled counterparts. The final two conditions combined the videos and corresponding audio, and their scrambled versions. No vocalization filtering or background noise cancellation was implemented to maintain the integrity of the vocal features.

As in our previous study^18^, we phase-scrambled the videos with a custom program, while vocalizations were time-domain scrambled^101^. This preserved their spectral content over longer time periods but removed structure at shorter timescales, rendering the vocalizations unintelligible^42^.

Each condition was incorporated in a block design task, wherein each block of stimuli lasted twelve seconds, interleaved with a fifteen-second baseline block. During the baseline block, a 0.36° circular black cue was displayed at the screen center against a gray background. Each run repeated the six conditions four times. To randomize the presentation of conditions in each run, we created eight different stimulus sets. These were counterbalanced within and between animals (Fig. 1e).

During the scanning sessions, monkeys were placed in a horizontal MR scanner (9.4 T) in a sphinx position within an MRI-compatible restraint system. Their heads were secured using a head post, and MRI-compatible auditory tubes were worn^42,61^. After performing the head fixation steps, the MRI-compatible auditory tubes (S14, Sensimetrics, Gloucester, MA) were directly placed into the animals’ ear canals bilaterally and were fixed using reusable sound-attenuating silicone earplugs (Amazon) and self-adhesive veterinary bandage (Fig. 1a)^62^.

An MR-compatible camera (model 12M-i, MRC Systems GmbH, Heidelberg, Germany) was positioned to monitor the animal during acquisition. Horizontal and vertical eye movements were tracked at a frequency of 60 Hz using a video eye tracker (ISCAN ETL-200 system, Boston, Massachusetts). Analysis of functional run data was performed using a custom R script. In each experimental condition and during baseline periods (i.e., fixation point in the center of the screen), the animals spent more than 79% of the time looking at the screen (baseline: 82.6%; marmoset face videos: 83.7%; vocalizations: 84.9%; marmoset face videos with vocalizations: 90.4%; scrambled marmoset face videos: 79.4%; scrambled vocalizations: 82.4%; scrambled marmoset face videos with corresponding scrambled vocalizations: 83.5%). Although a one-way ANOVA test indicated an effect of condition on viewing time (F(2.62,13.10) = 3.69, *p* = 0.044), this result was not robust after Bonferroni correction for multiple comparisons (minimum adjusted *p*-value = 0.58). Consequently, any differences in fMRI activation between the conditions cannot be attributed to differences in exposure to the stimuli.

Visual stimuli were projected onto a forward-facing plastic screen positioned 119 cm from the animal’s head using an LCSD-projector (Model VLP-FE40, Sony Corporation, Tokyo, Japan) via a back-reflection on a first surface mirror. We used Keynote software (version 12.0, Apple Incorporated, CA) for stimulus display. The onset of each stimulus was synchronized with an MRI TTL pulse triggered by a python program running on a Raspberry Pi (model 3B + , Raspberry Pi Foundation, Cambridge, UK). The animals received a reward only before and after each scanning session, not during sessions.

### fMRI acquisition and parameters

Imaging was performed at the Center for Functional and Metabolic Mapping at the University of Western Ontario. Data was collected using a 9.4 T/31 cm horizontal bore magnet and a Bruker BioSpec Avance III console running the Paravision-7 software package (Bruker BioSpin Corp). A custom-built gradient coil, with a 15 cm inner diameter and maximum gradient strength of 1.5 mT/m/A, coupled with eight separate receive channels was employed^61^ (Fig. 1a, b).

Eight functional images were acquired per animal over varying sessions, depending on each animal’s compliance. We utilized a gradient-echo based single-shot echo-planar images (EPI) sequence, with parameters set as follows: TR = 3 s, acquisition time TA = 1.5 s, TE = 15 ms, flip angle = 40°, field of view = 64 × 48 mm, matrix size = 96 × 128, isotropic resolution of 0.5 mm^3^, 42 axial slices, bandwidth = 400 kHz, and a GRAPPA acceleration factor of 2 (left-right). An additional set of EPIs, featuring an opposite phase-encoding direction (right-left), was collected for the EPI-distortion correction.

To diminish potential auditory stimuli masking by scanner noise, we employed a continuous acquisition paradigm that incorporated silent periods^42^. Despite the continuous presentation of auditory stimuli during each 12- second stimulus block, the scanner noise level turned off for 1.5-second periods within each 3-second TR. Consequently, we used a 3-second TR but collected  all slices within 1.5 s.

In each session per animal, we also acquired a T2-weighted structural image, with parameters set as follows: TR = 7 s, TE = 52 ms, field of view = 51.2 × 51.2 mm, resolution of 0.133 × 0.133 × 0.5 mm, 45 axial slices, bandwidth = 50 kHz, and a GRAPPA acceleration factor of 2.

### fMRI preprocessing

The data was processed using AFNI^102^ and FMRIB/FSL^103^ software packages. Initially, the raw functional images were converted into NifTI format using AFNI’s dcm2nixx function, and then reoriented from the sphinx position using FSL’s fslswapdim and fslorient functions. The functional images were despiked with AFNI’s 3Ddespike function, and volume were registered to the middle volume of each time series using AFNI’s 3dvolreg function. We stored the motion parameters from volume registration for later use with nuisance regression. Subsequently, functional images were smoothed using a full width at half-maximum Gaussian kernel (FWHM) of 1.5 mm with AFNI’s 3dmerge function and bandpass filtered from 0.1 to 0.01 Hz with AFNI’s 3dBandpass function. For each run, an average functional image was calculated and linearly registered to the respective T2-weighted anatomical image of each animal using FSL’s FLIRT function. For this process, the T2-weighted anatomical images were manually skull-stripped, and the mask of each animal was applied to the corresponding functional images. The transformation matrix obtained after the registration was used to transform the 4D time series data.

Lastly, the T2-weighted anatomical images were registered to the NIH marmoset brain atlas^104^ via nonlinear registration using Advanced Normalization Tools (ANTs’ ApplyTransforms function).

### fMRI statistical analysis

We employed the BLOCK function within AFNI’s 3dDeconvolve tool to model the hemodynamic response for each condition. This approach uses a predefined canonical HRF shape, parameterized to match our experimental design with 12 s stimulus blocks. These parameterized HRFs were used as regressors in a general linear model (GLM), complemented by polynomial detrending and motion parameters. This yielded six T-value maps per run for each subject, corresponding to our experimental conditions. These resultant regression coefficient maps were then registered to the NIH marmoset brain atlas template space^104^ using the transformation matrices obtained with the registration of anatomical images on the template (see above).

These maps were then subject to group level comparison via paired t-tests using AFNI’s 3dttest + + function, resulting in Z-value maps. To protect against false positives and control for multiple comparisons, we applied a clustering method derived from 10,000 Monte Carlo simulations to the resultant z-test maps using the ClustSim option (α = 0.05). This method involves setting a cluster-forming threshold of *p* < 0.01 uncorrected, followed by applying a family-wise error (FWE) correction of *p* < 0.05 at the cluster-level.

These Z-value maps were displayed on fiducial maps obtained from the Connectome Workbench (v1.5.0,^105^) using the NIH marmoset brain template^104^, and on coronal sections. The Paxinos parcellation^106^ of the NIH marmoset brain atlas^104^ was used to define anatomical locations of cortical and subcortical regions.

Initially, we identified voxels that exhibited significantly stronger activation during task engagement in each condition by contrasting each condition with the baseline period (i.e., marmoset face videos > baseline, scrambled marmoset face videos > baseline, vocalizations > baseline, scrambled vocalizations > baseline, marmoset face videos with vocalizations > baseline and scrambled marmoset face videos with scrambled vocalizations > baseline). To identify the areas involved in the processing of each modality (i.e., video, audio, and audiovisual conditions) and those common between modalities, we created a conjunction map between the three conditions, separately considering both intact and scrambled conditions. We used the AFNI 3dcalc-step function, inputting the thresholded resultant z-test maps obtained through paired t-tests (as described above). This allowed us to observe specific activations for each condition and the shared activations between conditions. Finally, to identify brain regions more activated by intact compared to scrambled stimuli, we contrasted the activations for marmoset face videos, vocalizations and marmoset face videos with corresponding vocalizations conditions against their scrambled versions (i.e., marmoset face videos > scrambled marmoset face videos, vocalizations > scrambled vocalizations, marmoset face videos with vocalizations > scrambled marmoset face videos with scrambled vocalizations). As previously, we also conducted a conjunction map between these three contrasts to observe specific and shared activations between the conditions.

Subsequently, we investigated the superadditive effect to determine the voxels more activated by combined audiovisual stimulation compared to the summed responses of unimodal auditory and visual stimulations. This comparison involved contrasting the multisensory condition (i.e., faces with corresponding vocalizations) with the summed response of the unimodal conditions (i.e., face videos plus vocalizations).

In order to visualize the response levels under the different conditions, we extracted 68 cortical ROIs from each hemisphere based on the Paxinos parcellation^106^ of the NIH marmoset brain atlas^104^. These ROIs were categorized according to their cortical position: 9 ROIs corresponded to visual areas, 7 to ventrolateral prefrontal and orbital frontal areas, 6 to dorsolateral prefrontal and premotor areas, 9 to posterior cingulate and medial prefrontal areas, 8 to posterior parietal areas, 8 to lateral, inferior, and ventral temporal areas, 13 to auditory areas, and 8 to the insula and other regions in the lateral sulcus. Beta values for each condition and run were extracted from the resultant regression coefficient maps using AFNI’s 3dmaskave function in each ROIs. Differences between conditions were computed using two-sided paired t-tests with false discovery rate (FDR) post-hoc correction (*p* < 0.05) using a custom-written Matlab script (R022a, The Mathworks). This analytical approach enabled us to conduct a detailed examination of response levels in specific cortical areas under the different auditory, visual, and audiovisual conditions. Additionally, within each ROIs, we computed the difference between the multisensory condition and the sum of the unisensory visual and auditory conditions to investigate the superadditive effect using two-sided paired t-tests (*p* < 0.05).

### Reporting summary

Further information on research design is available in the Nature Portfolio Reporting Summary linked to this article.

### Supplementary information

Peer Review File

Supplementary Information

Reporting Summary

## Data Availability

Data supporting this study are available on OSF at https://osf.io/e2h84/?view_only=b2454c28ab2344fea9f5ef650a7701bd.

## Code availability

The codes supporting this study are available on OSF at https://osf.io/e2h84/?view_only=b2454c28ab2344fea9f5ef650a7701bd.
