# Supplementary material for: Mapping of facial and vocal processing in common marmosets with ultra-high field fMRI
Source: Commun Biol. 2024 Mar 13;7:317. doi: 10.1038/s42003-024-06002-1 (PMC10937914; doi:10.1038/s42003-024-06002-1)
Supplement: Supplementary file 2 — Supplementary Information [file 42003_2024_6002_MOESM2_ESM.pdf]

Supplementary information

Supplementary Figure 1: Brain networks activated by each condition versus baseline for the right hemisphere.

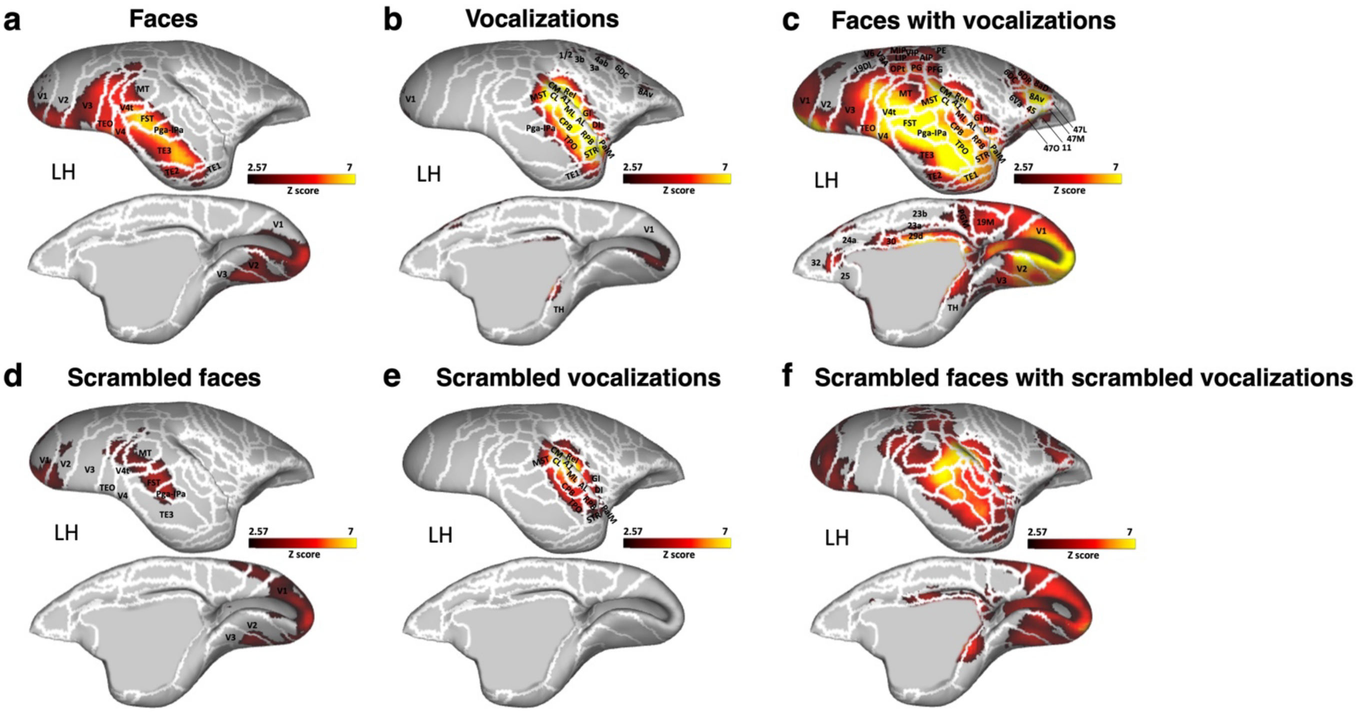

This figure represents the group functional map for each condition, showing significantly greater activations compared to baseline: marmoset face videos (**a**), marmoset vocalizations (**b**), marmoset face videos with corresponding vocalizations (**c**), scrambled marmoset face videos (**d**), scrambled marmoset vocalizations (**e**), and scrambled marmoset face videos with corresponding scrambled vocalizations (**f**). These group maps are based on data from six awake marmosets and are displayed on both lateral and medial views of the fiducial marmoset cortical surfaces, right hemisphere. The white line delineates the regions based on the Paxinos parcellation [63] of the NIH marmoset brain atlas [64]. The activation threshold corresponds to z-scores > 2.57 (p<0.01, AFNI's 3dttest++, cluster-size correction α=0.05 from 10000 Monte-Carlo simulations).

Supplementary Figure 2: Beta values across hemispheres for intact and scrambled conditions in 67 ROIs.

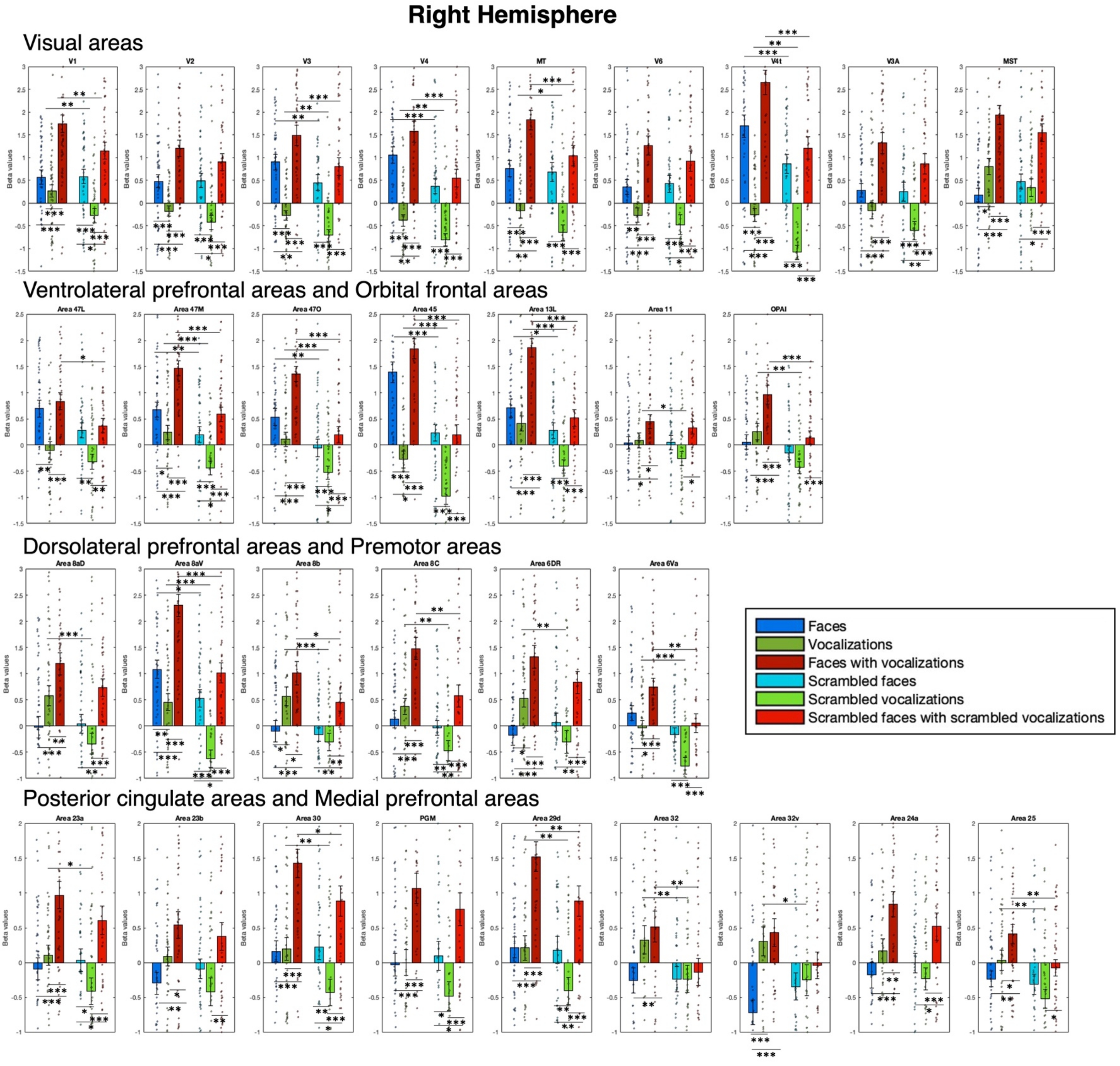

Right Hemisphere

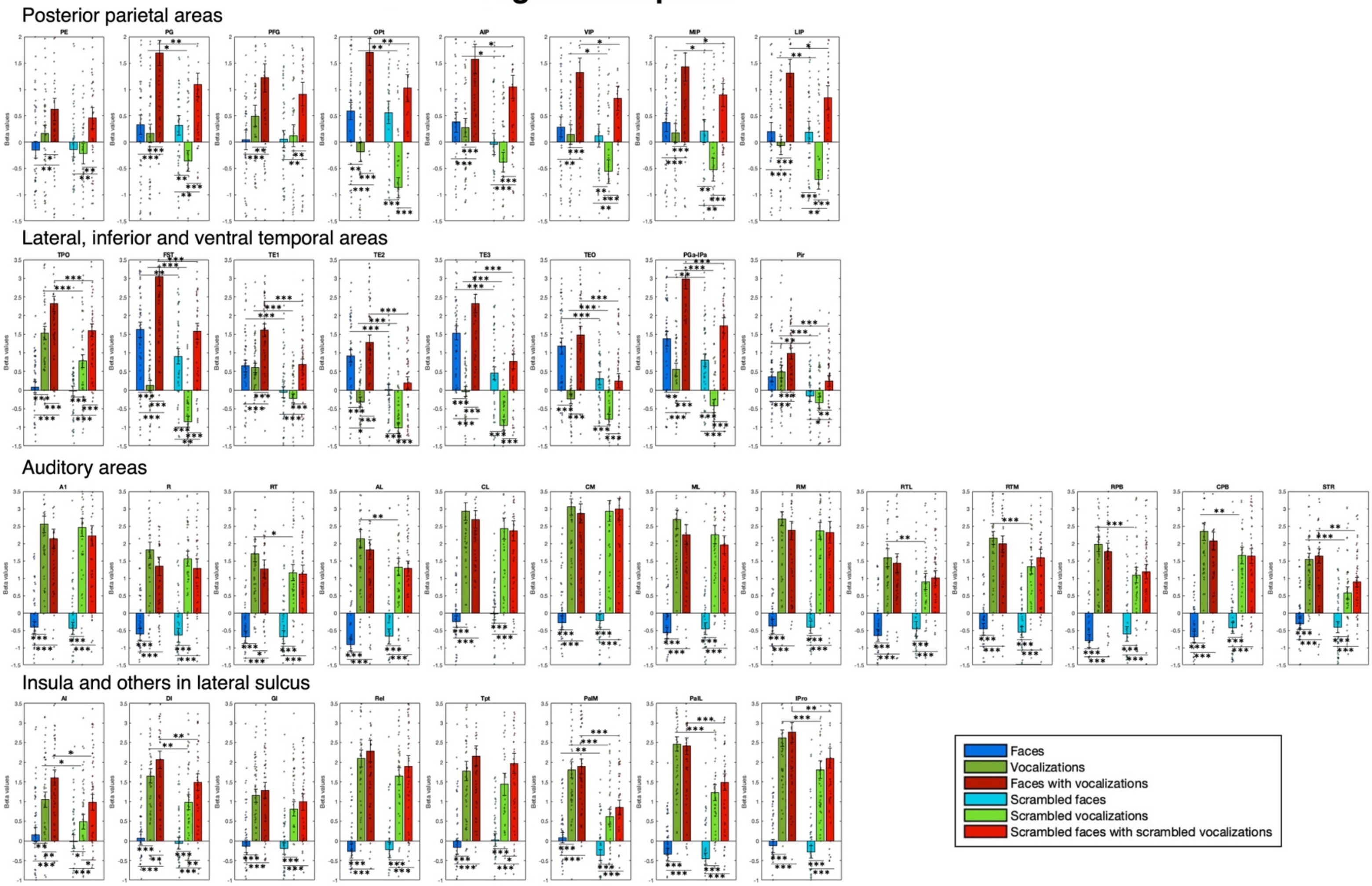

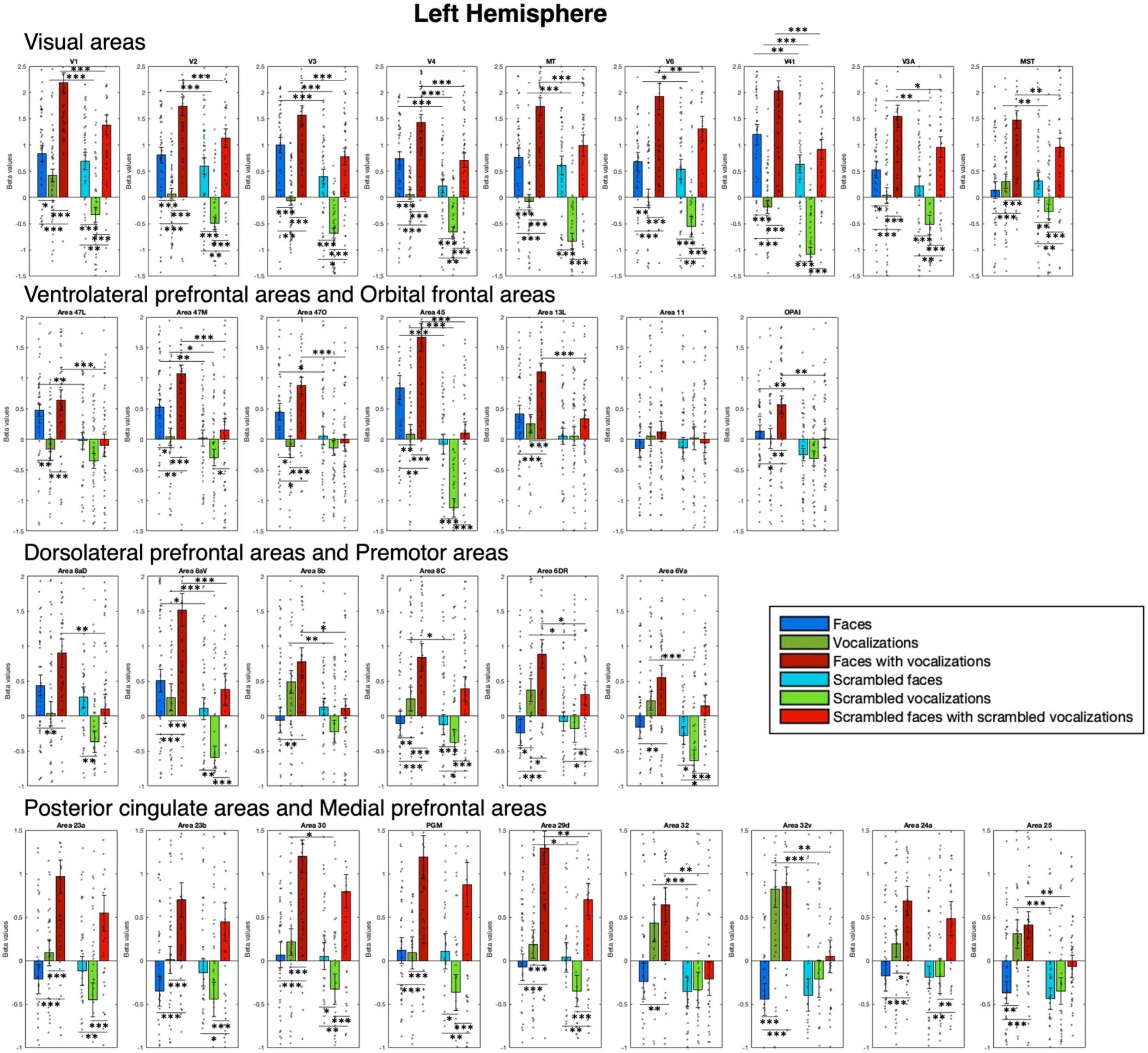
Left Hemisphere
Visual areas
V1
V2
V3
V4
MT
V6
V4t
V3A
MST
Ventrolateral prefrontal areas and Orbital frontal areas
Area 47L
Area 47M
Area 47O
Area 45
Area 13L
Area 11
OPAI
Dorsolateral prefrontal areas and Premotor areas
Area 8aD
Area 8aV
Area 8b
Area 8C
Area 6DR
Area 6Va
Posterior cingulate areas and Medial prefrontal areas
Area 23a
Area 23b
Area 30
PGM
Area 29d
Area 32
Area 32v
Area 24a
Area 25
Beta values
Faces
Vocalizations
Faces with vocalizations
Scrambled faces
Scrambled vocalizations
Scrambled faces with scrambled vocalizations

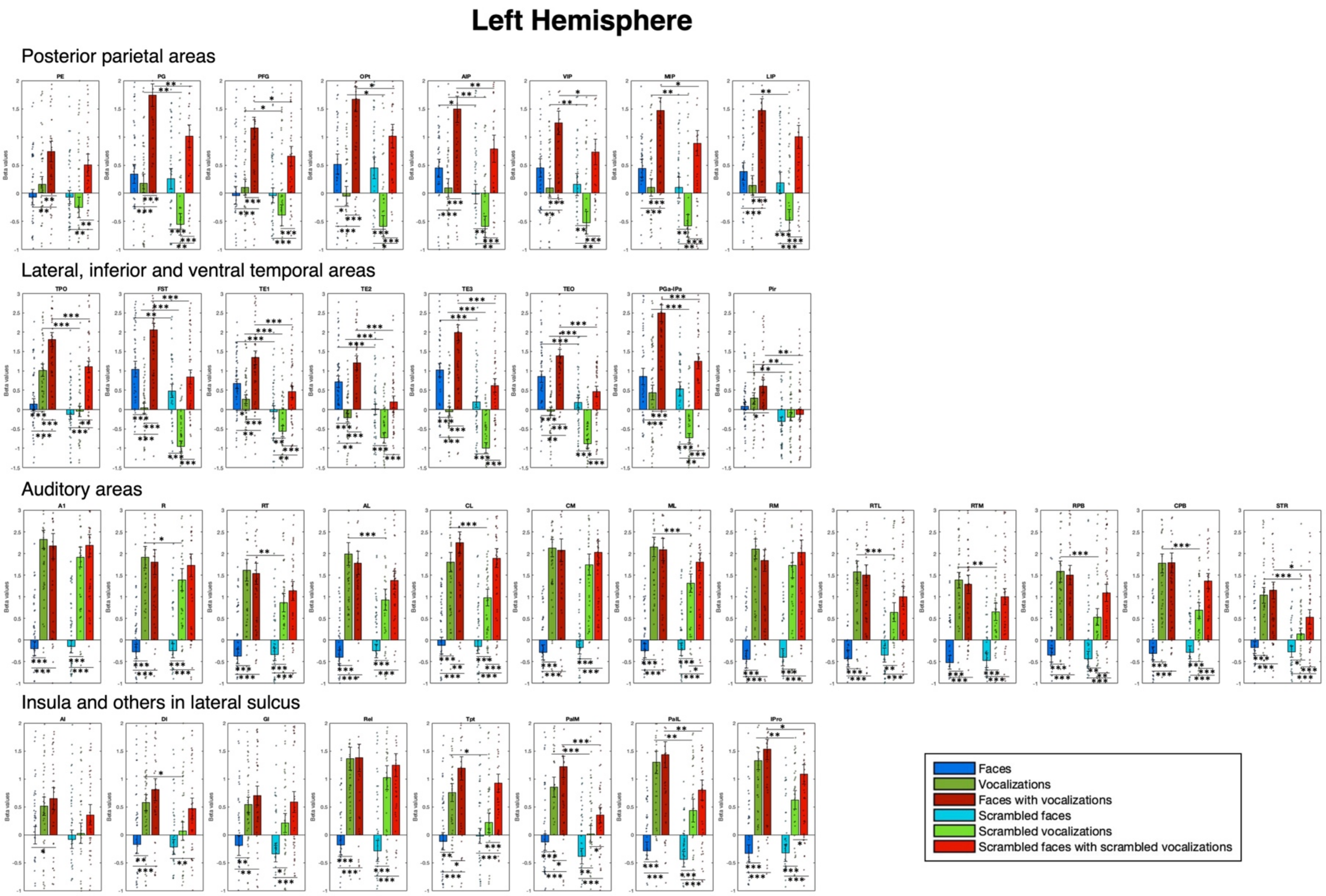

This figure presents a series of bar plots for the right and left hemispheres, illustrating beta values for each ROI under various conditions: marmoset faces (blue), vocalizations (green), marmoset faces with vocalizations (red), scrambled marmoset faces (cyan), scrambled vocalizations (light green), and scrambled marmoset faces with scrambled vocalizations (light red). The plots display mean beta values with error bars indicating the standard error of the mean (SEM). Horizontal bars with asterisks indicate significant differences between conditions, determined by two-sided paired t-tests with FDR correction for multiple comparisons: *$p < 0.05$, **$p < 0.01$, ***$p < 0.001$. ROIs are organized into distinct cortical groups: Visual areas, Ventrolateral prefrontal areas and orbital frontal areas, Dorsolateral prefrontal areas and premotor areas, Posterior cingulate areas and medial prefrontal areas, Posterior parietal areas, Lateral, inferior, and ventral temporal areas, Auditory areas, Insula and others in lateral sulcus.

Supplementary Figure 3: Brain activation patterns for marmoset vocalizations and scrambled vocalizations compared to baseline.

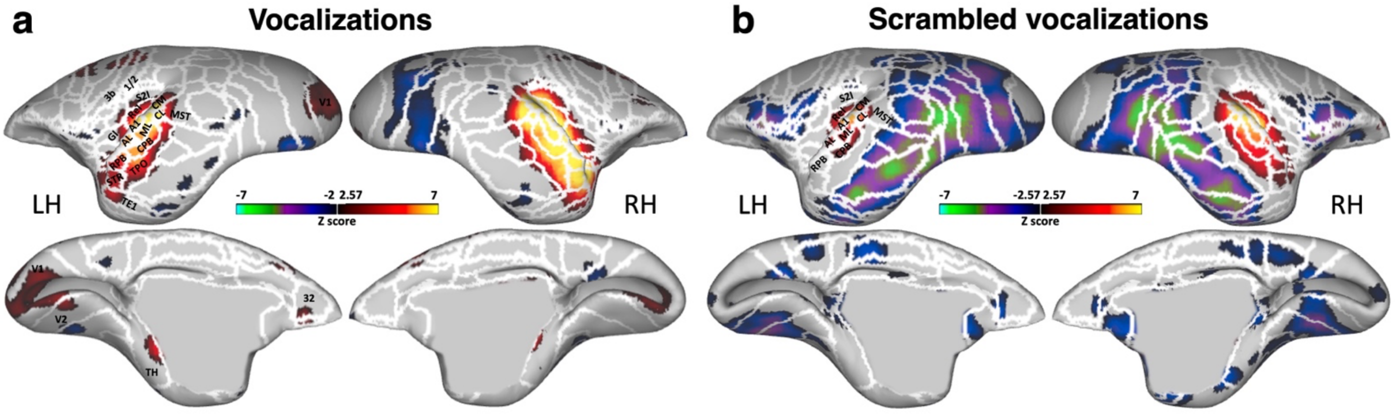

This figure displays the group functional maps for marmoset vocalizations (**a**) and scrambled vocalizations (**b**), each compared to baseline. The maps exhibit significant positive (red-yellow spectrum) and negative (blue-green spectrum) activations across both lateral and medial views of the marmoset cortical surfaces for the left and right hemisphere. Anatomical delineations follow the Paxinos parcellation of the NIH marmoset brain atlas. Activation thresholds are set at z-score > 2.57 (positive) or < -2.57 (negative), corresponding to p<0.01, with cluster-size correction applied α=0.05, derived from 10000 Monte-Carlo simulations in AFNI's 3dttest++.

Supplementary Table 1. Coordinates of the peak activation with Z value associated of each region obtained from the activation map for the condition marmoset vocalizations versus baseline.

Brain region	Hemisphere	Coordinates of peak activation			Peak z-value
		x	y	z	
V1	Right	3	-8	12	3.6
	Left	-3	-9	12	3.6
PFG	Right	8	5	16	3.12
	Left	-	-	-	-
TPt	Right	8	6	14	5.2
	Left	-9	6	13	5.05
MST	Right	10	6	12	6.3
	Left	-9	6	12	4.78
TPO	Right	10	6	11	6.4
	Left	-11	6	11	5.28
PGa - IPa	Right	11	6	9	4.03
	Left	-	-	-	-
S2I	Right	7	8	14	5.09
	Left	-	-	-	-
A1	Right	9	9	12	13
	Left	-10	8	12	13
CM	Right	9.1	8.1	11.4	13
	Left	-9.4	8.2	12.4	13
ML	Right	10	8	12	13
	Left	-10	8	12	13
AL	Right	11	9	10	8.04
	Left	-11	9	10	6.22
CPB	Right	10	8	10	7.80

	Left	-10	8	10	5.9
GI	Right	8	11	10	4.48
	Left	-9	11	10	3.62
R	Right	10	10	11	7.88
	Left	-10	10	11	6.25
RM	Right	9	10	10	13
	Left	-10	10	10	6.76
TPro	Right	9	10	9	13
	Left	-9	10	9	6.71
DI	Right	8	11	9	7.83
	Left	-9	11	8	4.89
RT	Right	9	11	10	7.52
	Left	-10	11	9	6.33
RTM	Right	11	7	6	
	Left	-9.9	10.6	8.9	6.82
PaIL	Right	9	11	7	13
	Left	-9	11	7	5.14
PaIM	Right	8	12	6	6.70
	Left	-8	12	6	4.42
Pir	Right	6	13	6	3.8
	Left	-6	13	5	2.77
STR	Right	9	12	7	7.80
	Left	-9	13	7	5.26
TPO	Right	10	8	8	6.7
	Left	-11	8	7	5.8

**TPPro**	**Right**	**8**	**13**	**6**	**6.55**
	**Left**	**-8**	**13**	**6**	**3.97**
**TE1**	**Right**	**8**	**11**	**5**	**6.72**
	**Left**	**-8**	**11**	**4**	**5.41**
**36**	**Right**	**7**	**11**	**4**	**3.87**
	**Left**	**-**	**-**	**-**	**-**
**ReI**	**Right**	**8**	**9**	**11**	**7.35**
	**Left**	**-8**	**9**	**11**	**6.88**
**IPro**	**Right**	**9**	**9**	**10**	**13**
	**Left**	**-9**	**9**	**10**	**7.33**
**Area 1/2**	**Right**	**5**	**9**	**17**	**3.03**
	**Left**	**-5**	**8**	**17**	**4.19**
**Area 3b**	**Right**	**5**	**9**	**17**	**3.27**
	**Left**	**-5**	**10**	**17**	**4.35**
**Area 3a**	**Right**	**2**	**9**	**18**	**3.25**
	**Left**	**-2**	**9**	**18**	**2.86**
**Area 4ab**	**Right**	**4**	**11**	**17**	**3.19**
	**Left**	**-5**	**11**	**17**	**3.5**
**Area 6DC**	**Right**	**3**	**15**	**16**	**4.08**
	**Left**	**-1**	**15**	**15**	**3.43**
**Area 6M**	**Right**	**1**	**15**	**16**	**4.27**
	**Left**	**-2**	**15**	**16**	**2.96**
**Area 8b**	**Right**	**2**	**16**	**15**	**4.58**
	**Left**	**-1**	**16**	**15**	**3.8**
**Area 8Av**	**Right**	**6**	**16**	**12**	**3.72**

	**Left**				
**Area 13L**	**Right**	**5**	**16**	**10**	**3.5**
	**Left**	**-**	**-**	**-**	**-**
**Area 24b**	**Right**	**-**	**-**	**-**	**-**
	**Left**	**-1**	**15**	**13**	**3.85**
**Area 32**	**Right**	**-**	**-**	**-**	**-**
	**Left**	**-1**	**18**	**10**	**3.74**
**Area 32v**	**Right**	**-**	**-**	**-**	**-**
	**Left**	**-1**	**18**	**9**	**4.16**
**Area 25**	**Right**	**-**	**-**	**-**	**-**
	**Left**	**-1**	**16**	**10**	**3.02**
