# Supplementary material for: Mapping of facial and vocal processing in common marmosets with ultra-high field fMRI
Source: Commun Biol. 2024 Mar 13;7:317. doi: 10.1038/s42003-024-06002-1 (PMC10937914; doi:10.1038/s42003-024-06002-1)
Supplement: Supplementary file 3 — Reporting Summary [file 42003_2024_6002_MOESM3_ESM.pdf]

nature portfolio

Corresponding author(s): Audrey Dureux

Last updated by author(s): Feb 28, 2024

Reporting Summary

Nature Portfolio wishes to improve the reproducibility of the work that we publish. This form provides structure for consistency and transparency in reporting. For further information on Nature Portfolio policies, see our Editorial Policies and the Editorial Policy Checklist.

Statistics

For all statistical analyses, confirm that the following items are present in the figure legend, table legend, main text, or Methods section.

n/a	Confirmed	
☐	☒	The exact sample size (*n*) for each experimental group/condition, given as a discrete number and unit of measurement
☐	☒	A statement on whether measurements were taken from distinct samples or whether the same sample was measured repeatedly
☐	☒	The statistical test(s) used AND whether they are one- or two-sided<br>*Only common tests should be described solely by name; describe more complex techniques in the Methods section.*
☒	☐	A description of all covariates tested
☐	☒	A description of any assumptions or corrections, such as tests of normality and adjustment for multiple comparisons
☐	☒	A full description of the statistical parameters including central tendency (e.g. means) or other basic estimates (e.g. regression coefficient) AND variation (e.g. standard deviation) or associated estimates of uncertainty (e.g. confidence intervals)
☐	☒	For null hypothesis testing, the test statistic (e.g. *F*, *t*, *r*) with confidence intervals, effect sizes, degrees of freedom and *P* value noted<br>*Give P values as exact values whenever suitable.*
☒	☐	For Bayesian analysis, information on the choice of priors and Markov chain Monte Carlo settings
☒	☐	For hierarchical and complex designs, identification of the appropriate level for tests and full reporting of outcomes
☒	☐	Estimates of effect sizes (e.g. Cohen's *d*, Pearson's *r*), indicating how they were calculated

Our web collection on statistics for biologists contains articles on many of the points above.

Software and code

Policy information about availability of computer code

Data collection	Scanner Software: Paravision-7 software package by Bruker BioSpin Corp.<br>Eye Movement Tracking Software: Acqnowledge (Version 8.1.22) associated with ISCAN ETL-200 system.
Data analysis	Processing and Analysis: AFNI (Version AFNI_22.0.15) and FMRIB/FSL (Version 6.0.5.1).<br>Conversion to NifTI: AFNI's dcm2nixx function.<br>Noise and Artifact Removal: AFNI's 3dToutcount, 3dDespike, 3dTshift, and 3dvolreg functions.<br>Statistical Modeling & Inference: AFNI's 3dDeconvolve, 3dBandpass, and 3dttest++ functions.<br>Normalization: Advanced Normalization Tools (ANTs) with the ApplyTransforms function.<br>Eye tracking Data Analysis: R studio (Version 2023.06.0+421).

For manuscripts utilizing custom algorithms or software that are central to the research but not yet described in published literature, software must be made available to editors and reviewers. We strongly encourage code deposition in a community repository (e.g. GitHub). See the Nature Portfolio guidelines for submitting code & software for further information.

Data

Policy information about availability of data

All manuscripts must include a data availability statement. This statement should provide the following information, where applicable:

- Accession codes, unique identifiers, or web links for publicly available datasets
- A description of any restrictions on data availability
- For clinical datasets or third party data, please ensure that the statement adheres to our policy

Data supporting this study are available on OSF at https://osf.io/e2h84/?view_only=b2454c28ab2344fea9f5ef650a7701bd.

Research involving human participants, their data, or biological material

Policy information about studies with human participants or human data. See also policy information about sex, gender (identity/presentation), and sexual orientation and race, ethnicity and racism.

Reporting on sex and gender	No human participants involved in the study.
Reporting on race, ethnicity, or other socially relevant groupings	No human participants involved in the study.
Population characteristics	No human participants involved in the study.
Recruitment	No human participants involved in the study.
Ethics oversight	No human participants involved in the study.

Note that full information on the approval of the study protocol must also be provided in the manuscript.

Field-specific reporting

Please select the one below that is the best fit for your research. If you are not sure, read the appropriate sections before making your selection.

☒ Life sciences ☐ Behavioural & social sciences ☐ Ecological, evolutionary & environmental sciences

For a reference copy of the document with all sections, see nature.com/documents/nr-reporting-summary-flat.pdf

Life sciences study design

All studies must disclose on these points even when the disclosure is negative.

Sample size	The sample size for the fMRI study, comprising 6 common marmosets, was not predetermined through power analysis but was instead determined by the availability of experimental animals in our laboratory. We believe this sample size is adequate to validate our hypotheses, given that many other fMRI studies involving common marmosets have employed similar or smaller sample sizes.
Data exclusions	During the fMRI study, the investigator monitored and noted the animal's compliance in real-time for each run. Any runs where the animal closed its eyes for two or more stimulation blocks, irrespective of the experimental condition, were excluded from the analyses.
Replication	We assessed the quality of the signal across sessions and monkeys by calculating the signal-to-noise ratio (SNR) for each functional run. A low SNR might indicate improper coil positioning, potentially yielding poor or noisy results. However, the SNR for runs chosen in our fMRI experiment remained consistent and comparable across both sessions and individual monkeys.
Randomization	No randomization was conducted as participants were part of a single experimental group.
Blinding	Blinding was not implemented as there was no randomization; all participants were part of a single experimental group.

Reporting for specific materials, systems and methods

We require information from authors about some types of materials, experimental systems and methods used in many studies. Here, indicate whether each material, system or method listed is relevant to your study. If you are not sure if a list item applies to your research, read the appropriate section before selecting a response.

Materials & experimental systems

n/a	Involved in the study
☒	☐ Antibodies
☒	☐ Eukaryotic cell lines
☒	☐ Palaeontology and archaeology
☐	☒ Animals and other organisms
☒	☐ Clinical data
☒	☐ Dual use research of concern
☒	☐ Plants

Methods

n/a	Involved in the study
☒	☐ ChIP-seq
☒	☐ Flow cytometry
☐	☒ MRI-based neuroimaging

Animals and other research organisms

Policy information about studies involving animals; ARRIVE guidelines recommended for reporting animal research, and Sex and Gender in Research

Laboratory animals	Six common marmosets (Callithrix jacchus) participated in the awake fMRI study. This included two females (weight: 315 and 150 g; age: 44 months) and four males (weight range: 365-459 g; age range: 32-44 months).
Wild animals	No wild animals were involved in this study.
Reporting on sex	The experiment included an unequal number of male and female marmosets. With only two females tested, it's not feasible to conduct a separate sex-based analysis. Thus, all analyses presented in the manuscript treat the sample as a whole, without differentiation by sex.
Field-collected samples	The study did not involve samples collected from the field.
Ethics oversight	All experimental procedures complied with the Canadian Council of Animal Care guidelines and were approved under protocol #2021-111 by the Animal Care Committee of the University of Western Ontario Council on Animal Care.

Note that full information on the approval of the study protocol must also be provided in the manuscript.

Plants

Seed stocks	NA.
Novel plant genotypes	NA.
Authentication	NA.

Magnetic resonance imaging

Experimental design

Design type	Task-based fMRI utilizing a block design.
Design specifications	Our experiment employed six distinct stimuli types: two video conditions, two audio conditions, and two conditions that combined both video and corresponding audio. Specifically:<br><br>The video conditions displayed marmoset face videos (without sound) and their scrambled versions.<br>The audio conditions featured vocalizations extracted from the videos and their scrambled versions.<br>The combination conditions presented the marmoset face videos with their corresponding vocalizations both in their original and scrambled forms.<br>We used a block design task for the experiment. Each stimulus block lasted twelve seconds and was interleaved with a fifteen-second baseline block, during which a circular black cue was centrally displayed against a gray background. Each run presented the six conditions four times, resulting in 24 stimulus blocks interspersed with 23 baseline blocks. Consequently, the total duration of each run was 663 seconds.
Behavioral performance measures	The experiment involved a free-viewing task where videos were presented to awake, head-fixed animals. To confirm the monkey's engagement, we calculated the percentage of time looking at the screen in every run. For that, we tracked

horizontal and vertical eye movements at a frequency of 60Hz utilizing a video eye tracker. Subsequently, a one-way ANOVA was conducted to ascertain if any experimental conditions had any significant impact on the viewing duration.

Acquisition

Imaging type(s)

Functional.

Field strength

9.4T.

Sequence & imaging parameters

Functional Imaging (EPI):

Sequence Type: Gradient-echo single-shot echo-planar images (EPI)
TR: 3s
Acquisition Time (TA): 1.5s
TE: 15ms
Flip Angle: 40°
Field of View: 64x48 mm
Matrix Size: 96x128
Resolution: Isotropic 0.5 mm^3
Slices: 42 axial slices
Bandwidth: 400 kHz
GRAPPA Acceleration Factor: 2 (left-right)
For EPI-distortion correction, we acquired an additional set of EPIs with an opposite phase-encoding direction (right-left).

To mitigate potential auditory stimulus masking by the scanner noise, we adopted a continuous acquisition paradigm with silent periods. This ensured that, even though auditory stimuli continuously played during each 12-second stimuli block, the scanner noise level ceased during 1.5-second intervals within every 3-second TR. As a result, while employing a 3-second TR, all slices were acquired in a span of 1.5 seconds.

Structural Imaging (T2-weighted):

TR: 7s
TE: 52ms
Field of View: 51.2x51.2 mm
Resolution: 0.133x0.133x0.5 mm
Slices: 45 axial slices
Bandwidth: 50 kHz
GRAPPA Acceleration Factor: 2.

Area of acquisition

Whole brain scan.

Diffusion MRI ☐ Used ☒ Not used

Preprocessing

Preprocessing software

We employed the AFNI and FMRIB/FSL software packages for data preprocessing.

1. Conversion and Reorientation:
Raw functional images were converted into NifTI format using AFNI's dcm2nixx function.
Images were then reoriented from the sphinx position using FSL's fslswapdim and fslorient functions.

2. Despiking and Volume Registration:
The functional images were despiked with AFNI's 3Ddespike function.
Volumes were registered to the middle volume of each time series using AFNI's 3dvolreg function.
Motion parameters from volume registration were stored for later nuisance regression.

3. Smoothing and Bandpass Filtering:
Functional images were smoothed using a full width at half-maximum Gaussian kernel (FWHM) of 1.5mm with AFNI's 3dmerge function.
Images were bandpass filtered from 0.1 to 0.01 Hz with AFNI's 3dBandpass function.

4. Registration to Anatomical Images:
An average functional image for each run was calculated and linearly registered to its corresponding T2-weighted anatomical image using FSL's FLIRT function. For this, the T2-weighted anatomical images were manually skull-stripped, and the mask of each animal was applied to the respective functional images.
The transformation matrix from the registration was used to transform the 4D time series data.

5. Atlas Registration:
T2-weighted anatomical images were registered to the NIH marmoset brain atlas using nonlinear registration via Advanced Normalization Tools (ANTs' ApplyTransforms function).

Normalization

For accurate comparisons and subsequent analysis, it was essential to align individual anatomical and functional images of the marmosets to a standardized reference. We chose the NIH marmoset brain atlas as this reference.

The registration to the atlas was achieved using nonlinear registration techniques, which provide more flexibility in aligning images than linear methods. Specifically, we employed the ApplyTransforms function available in the ANTs (Advanced Normalization Tools) package to execute this nonlinear registration.

Normalization template

NIH marmoset brain atlas (Liu et al., 2018).

Noise and artifact removal

The functional images, after reorientation, underwent a series of preprocessing steps to reduce noise and artifacts. Outliers in the data were identified and eliminated using AFNI's 3dToutcount. The data was then despiked using 3dDespike and time-corrected with 3dTshift. The motion parameters obtained from 3dvolreg from AFNI were preserved for subsequent use in nuisance regression to minimize the influence of motion-related artifacts in the analyses.

Volume censoring

No volume censoring performed.

Statistical modeling & inference

Model type and settings

We employed AFNI's 3dDeconvolve function to compute the hemodynamic response function (HRF) for each run. The task timings were convolved with the time series data using AFNI's 'BLOCK' convolution to derive the HRF estimates for each of the six experimental conditions. This approach uses a predefined canonical HRF shape, parameterized to match our experimental design with 12-second stimulus blocks. These parameterized HRFs were used as regressors in a general linear model (GLM), complemented by polynomial detrending regressors and the earlier mentioned motion parameters. This yielded six T-value maps for every animal per run, corresponding to our experimental conditions. The coefficient maps obtained from this regression were registered to the NIH marmoset brain atlas template space using the pre-obtained transformation matrices from the anatomical to template registration.

For group-level comparisons, we subjected these maps to paired t-tests via AFNI's 3dttest++ function, yielding Z-value maps. To protect against false positives and control for multiple comparisons, we applied a clustering approach based on 10,000 Monte Carlo simulations to the derived z-test maps utilizing the ClustSim option ($\alpha=0.05$). This technique established a preliminary threshold of $p<0.01$ (uncorrected), followed by the imposition of a family-wise error (FWE) correction at $p<0.05$ for cluster-level significance.

Effect(s) tested

1. Single Condition Activation vs. Baseline: First, we identified voxels showing significant activation during task engagement for each condition when compared to the baseline. The comparisons made were:
Marmoset face videos vs. baseline
Scrambled marmoset face videos vs. baseline
Vocalizations vs. baseline
Scrambled vocalizations vs. baseline
Marmoset face videos with vocalizations vs. baseline
Scrambled marmoset face videos with scrambled vocalizations vs. baseline

2. Conjunction Analysis Between Modalities: For discerning the areas integral to processing each modality (video, audio, and audiovisual) and those common across modalities, we generated a conjunction map between the three conditions. This was done separately for both intact and scrambled conditions. We employed AFNI's 3dcalc-step function, using the thresholded z-test maps from the paired t-tests as inputs. This revealed both unique and shared activations across conditions.

3. Intact vs. Scrambled Stimuli: To identify brain areas with higher activation for intact stimuli compared to scrambled versions, we contrasted the activations for marmoset face videos, vocalizations and marmoset face videos with corresponding vocalizations conditions against their scrambled versions (i.e., marmoset face videos > scrambled marmoset face videos, vocalizations > scrambled vocalizations, marmoset face videos with vocalizations > scrambled marmoset face videos with scrambled vocalizations). As previously, we also conducted a conjunction map between these three contrasts to observe specific and shared activations between the conditions.

4. Superadditive Effect Exploration: We delved into the superadditive effect by identifying voxels that were more activated by combined audiovisual stimulation than by the sum of unimodal auditory and visual stimulations. Specifically, we contrasted the multisensory condition (faces paired with vocalizations) against the sum of the unimodal conditions (face videos conditions plus vocalizations conditions).

5. ROI analysis: In order to visualize the response levels under the different conditions, we extracted 68 cortical ROIs from each hemisphere based on the Paxinos parcellation of the NIH marmoset brain atlas. Beta values for each condition and run were extracted from the resultant regression coefficient maps using AFNI's 3dmaskave function in each ROIs. Differences between conditions were computed using two-sided paired t-tests with FDR post-hoc correction ($p < .05$). This analytical approach enabled us to conduct a detailed examination of response levels in specific cortical areas under the different auditory, visual, and audiovisual conditions. Additionally, within each ROIs, we computed the difference between the multisensory condition and the sum of the unisensory visual and auditory conditions to investigate the superadditive effect using two-sided paired t-tests ($p < .05$).

Specify type of analysis: ☐ Whole brain ☐ ROI-based ☒ Both

Anatomical location(s)

We extracted 68 cortical ROIs from each hemisphere based on the Paxinos parcellation of the NIH marmoset brain atlas. These ROIs were categorized according to their cortical position: 9 ROIs corresponded to visual areas, 7 to ventrolateral prefrontal and orbital frontal areas, 6 to dorsolateral prefrontal and premotor areas, 9 to posterior cingulate and medial prefrontal areas, 8 to posterior parietal areas, 8 to lateral, inferior, and ventral temporal areas, 13 to auditory areas, and 8 to the insula and other regions in the lateral sulcus.

Statistic type for inference<br>(See Eklund et al. 2016)	We employed a cluster-wise analysis using AFNI's 3dttest++ in conjunction with the Clustsim option. An initial threshold of $p<0.01$ was used for cluster formation. The family-wise error rate was controlled at $\alpha=0.05$. The nearest-neighbor clustering method was applied, with a two-sided approach, using a 1-nearest-neighbor criterion.
Correction	To protect against false positives and control for multiple comparisons, we applied a clustering method derived from 10,000 Monte Carlo simulations to the resultant z-test maps using the ClustSim option ($\alpha=0.05$). This method involves setting a cluster-forming threshold of $p<0.01$ uncorrected, followed by applying a family-wise error (FWE) correction of $p<0.05$ at the cluster-level.

Models & analysis

n/a	Involved in the study
☒	☐ Functional and/or effective connectivity
☒	☐ Graph analysis
☒	☐ Multivariate modeling or predictive analysis
