# Supplementary material for: Mapping of facial and vocal processing in common marmosets with ultra-high field fMRI
Source: Commun Biol. 2024 Mar 13;7:317. doi: 10.1038/s42003-024-06002-1 (PMC10937914; doi:10.1038/s42003-024-06002-1)
Supplement: Supplementary file 1 — Peer Review File [file 42003_2024_6002_MOESM1_ESM.pdf]

nature portfolio

Peer Review File

Mapping of Facial and Vocal Processing in Common Marmosets with ultra-high field fMRI

Open Access This file is licensed under a Creative Commons Attribution 4.0 International License, which permits use, sharing, adaptation, distribution and reproduction in any medium or format, as long as you give appropriate credit to the original author(s) and the source, provide a link to the Creative Commons license, and indicate if changes were made. In the cases where the authors are anonymous, such as is the case for the reports of anonymous peer reviewers, author attribution should be to 'Anonymous Referee' followed by a clear attribution to the source work. The images or other third party material in this file are included in the article's Creative Commons license, unless indicated otherwise in a credit line to the material. If material is not included in the article's Creative Commons license and your intended use is not permitted by statutory regulation or exceeds the permitted use, you will need to obtain permission directly from the copyright holder. To view a copy of this license, visit http://creativecommons.org/licenses/by/4.0/.

Reviewers' comments:

Reviewer #1 (Remarks to the Author):

This is a well written manuscript, and I have no issues with the scientific content. My comments below are just suggestions.

a. Introduction, L67 and more generally, in the discussion. The authors seem to be speaking specifically about audiovisual integration of facial or social cues. There is also some literature about audiovisual integration of object/ sound source location, which seems to depend on a different set of structures. For example, in the macaque and marmoset the caudal belt and superior temporal poly sensory areas project directly to the primary visual cortex, which has been hypothesised to facilitate stimulus localisation in the periphery (Falchier et al. 2002; Majka et al. 2019). I would make sure that the focus is explicit in the introduction, and make the point in the Discussion that other forms of audiovisual integration are likely to depend on other sets of areas.

b. Results, L104. An intriguing observation is that the LGN is recruited by face videos, but not scrambled faces. Given that this nucleus is usually regarded as a low-level representation where cells analyse small parts of the visual field, this deserves some discussion. Perhaps feedback connections from the cortex could cause this effect?

c. Results, L116. the Paxinos parcellation of the NIH marmoset brain atlas - perhaps citation of the original work would be appropriate, given that this is where the criteria for area identification are given.

d. Results, L 128-134. The activations in non-auditory areas such as V1, V2, and primary somatosensory cortex are a bit surprising. It would be useful to have some quantification of the level of these activations, compared to "expected" activations in auditory areas. The color scales in the figures are a bit too compressed to give this information accurately, so a table with the sizes of the effects could help the reader evaluate better the relative magnitudes of the activations.

e. L130. In the Paxinos et al. atlas the superior temporal rostral area (STR) is included in the auditory cortex, being regarded as a likely high-order auditory area based on comparison with Pandya's work in the macaque. In addition, previous research in marmoset (de La Mothe et al. 2006; Burman et al. 2011) has argued for the areas just medial to the medial belt to be regarded as parts of an "extended" auditory cortex. This was originally referred to the proisocortical auditory area by the above authors, but has since been subdivided in the Paxinos atlas (IPro, PaIM, PaIL). Some of these areas show activations by the auditory stimuli. Thus, related to point (d) above, I would be interested to know the size of the effect detected there, relative to other areas.

f. P138. Was a specific part of area MST was activated? This question comes from the suggestion advanced by Majka et al. 2019, that area MST as defined in the marmoset atlas may include parts of the superior temporal polysnesory area (TPOc). If the activation was specifically located to the rostral part of MST this could be interesting; if not, please ignore this comment.

g. Regarding area 32: Reser et al. (2009) made the observation that the marmoset rostrotemporal area (RT) has a direct projection to what he considered "a caudal projection of area 10", based on Price's macaque map. From the section illustrated, this is most likely what is now regarded part of area 32. This would provide additional support to the idea that this region has a auditory cognition role.

Reviewer #2 (Remarks to the Author):

In this study, the authors investigated multisensory face and voice processing in marmosets using ultra-high field fMRI. By comparing brain response to face stimuli only, voice stimuli only, and multisensory face-voice stimuli, the authors found multisensory stimuli activated additional fronto-parietal networks beyond unimodal face and voice areas. The experiment is well-designed and carefully conducted, yet essential concerns must be addressed before the study can be published. Major and additional points are listed below.

Major points:
1. The process of unimodal and multisensory visual and auditory information has been extensively studied in literature, as the authors cited in the manuscript. Therefore, the novelty of the current study could be clearer—moreover, the motivation and significance of using marmosets as subjects need to be laid out.
2. The scrambled conditions are supposed to be control stimuli. However, they need to be adequately analyzed as controls. Specifically, the actual "face patch" and "voice patch" should derive from contrasting intact vs. scrambled conditions. The actual "multisensory face-voice area" should derive from contrasting intact vs. scrambled multisensory stimuli (as the authors did). Then, all comparisons between multisensory and unisensory conditions should be based on these scramble-controlled images. Without proper control of the scrambled conditions, it is unclear if areas respond to simple visual and auditory stimulation or higher-level communication stimuli.
3. The stimuli used in the experiment involve negative facial expressions, and negative cues tend to elicit more robust responses (as the authors discussed in the Introduction). The authors should discuss the results from this perspective.

Other points:
1. In fMRI analysis, the authors first estimated the timing and amplitude of each condition and then used the estimated HRF as the regressor. What is the rationale for this approach？ And is it circular? Why not just use the standard HRF, or model-free methods?
2. The influence of the multisensory process on unimodal areas would be interesting to investigate. E.g., would multisensory stimuli simply activate more areas beyond unimodal patches or activate more areas AND increase the activity in unimodal areas?
3. Relatedly, adding bar graphs showing the response level of different ROIs under different conditions would help demonstrate the superadditive effect (and when areas do not show a superadditive effect, how their responses vary across conditions).
4. Only the left hemisphere is shown in Figure 1, whereas the rest shows both hemispheres. It would be good to be consistent.

Reviewer #3 (Remarks to the Author):

This study investigates the integration of auditory and visual vocalizations in the marmoset brain using fMRI at 9.4T. By comparing brain-wide responses to face movies alone, acoustic vocalizations alone, and their combination, the authors reveal a broad and apparently robust network of cortical and subcortical areas whose activation enhanced by, or exclusive to, multimodal stimulation. The authors link their findings in the marmoset to those in humans and macaques, highlighting prefrontal cortical areas that may be involved in complex social perception.

I really like this paper, as the methods are impressive, the design is straightforward, the results are described succinctly, and the findings are striking. There is little to criticize. I list a few points below that the authors may want to take into consideration.

Points to consider:

The authors jump right into the results in Figure 1, perhaps because they have previously described their fMRI methods extensively in other papers. I did, however, find it somewhat wanting for a bit more description about the block design, fMRI brain coverage, analysis assumptions etc. Some readers at this point in the paper might look at the Methods to discover Figure 5, but I did not. If it were me, I would spend a figure on the methods, including some aspects of the imaging at 9.4T, which I think is a strength of this study, rather than a liability. Also, I would show off the fact that there are 6 subjects, novel methods, etc in supplemental material. I don't think any of this will lead to confusion that this is a methods paper. Just a suggestion.

The authors mention area 32 quite a bit, pointing to its role in processing coherent stimuli. That's interesting, but there's not much to grab onto there and I found the discussion essentially a restatement of the results.

A couple of other areas might also deserve mention. For example, is it known that the premotor areas respond exclusively to auditory stimuli (Fig 3A, showing this is only true for intact acoustic vocalizations)? Might that be related, for example, to directing the orientation toward a vocalizing conspecific (before the face is visualized? Also, the very strong multimodal enhancement of subcortical structures (SC, Pul), and peripheral visual cortex (e.g. calcarine V1) was striking. Somehow the combination of cues leads the brain to resonate with activity in a way that may not fit cleanly into how people traditionally think about "multimodal integration". The authors might want to make a bigger deal about this. It's not surprising that, as they say on p.12, "multisensory processing is not a simple summation of each modality but also involves additional brain regions". But this result seems like much more significant for understanding the integration of social sensory information than a refutation of that standard null hypotheses about additivity.

Reviewers' comments:

Reviewer #1 (Remarks to the Author):

This is a well written manuscript, and I have no issues with the scientific content. My comments below are just suggestions.

We are grateful for the reviewer's positive comments on our manuscript and appreciate the suggestions provided. We have carefully considered each recommendation and have made corresponding changes to the manuscript. We believe these changes have improved the clarity and impact of our work.

a. Introduction, L67 and more generally, in the discussion. The authors seem to be speaking specifically about audiovisual integration of facial or social cues. There is also some literature about audiovisual integration of object/ sound source location, which seems to depend on a different set of structures. For example, in the macaque and marmoset the caudal belt and superior temporal poly sensory areas project directly to the primary visual cortex, which has been hypothesised to facilitate stimulus localisation in the periphery (Falchier et al. 2002; Majka et al. 2019). I would make sure that the focus is explicit in the introduction, and make the point in the Discussion that other forms of audiovisual integration are likely to depend on other sets of areas.

We appreciate the insightful comments of the reviewer regarding the scope of our study. Indeed, our research specifically focuses on the audiovisual integration of facial or social cues in marmosets. We recognize that there is a substantial body of literature addressing audiovisual integration related to other types of stimuli like object and sound source localization, which likely involves different neural structures than those implicated in the integration of social signals.

In response to the suggestion, we refined the sentence in our Introduction to clearly delineate the scope of our investigation on the integration of facial expressions and vocalizations to explicit clarification on the specific domain of audiovisual integration we are exploring.

Furthermore, we agree with the suggestion to discuss in the manuscript that audiovisual integration can be multifaceted, with different forms depending on distinct neural substrates. To this end, we added to the Discussion section a paragraph acknowledging the literature on audiovisual integration for other types of stimuli, which can be different compared to the facial and social cue integration our study addresses.

These modifications can be found in the revised manuscript on page 4 of the introduction and on pages 34-35 in the discussion section, as follow:

Page 4: "Recent studies have begun to elucidate the neural substrates of multisensory integration in primates, demonstrating that audiovisual integration of social cues—specifically, faces and vocalizations—occurs in particular regions of the monkey face-patch and voice-patch systems 44–47 as well as in the ventral lateral prefrontal cortex (VLPFC) 48–50. Human studies have also

highlighted the involvement of temporal [51,52], frontal [53,54], and parietal [53] cortices in the processing of combined visual and auditory social information."

Pages 34-35: "While our study focuses on the integration of audiovisual social cues, it is important to note that other forms of audiovisual integration likely involve different neural substrates. A recent meta-analysis summarizing 121 human neuroimaging studies that examined the neural basis of audiovisual integration in humans, has demonstrated that the experimental context and stimulus complexity influence the brain networks identified during audio visual integration [95]. Their results suggest that audiovisual integration can occur via engaging a network of different brain regions at multiple levels which are highly context-dependent. These include sensory sites (i.e., middle and inferior occipital gyrus, fusiform gyrus, lingual gyrus, and the middle portion of superior temporal gyrus), subcortical sites (i.e., thalamus), and higher association sites (i.e., superior temporal cortex and middle and superior frontal gyrus). These studies indicate that the neural pathways for audiovisual integration appear to be flexible rather than a fixed network of brain regions, with superior temporal cortex playing a central role in these neural assemblies. Furthermore, in macaques and marmosets, audiovisual integration for object/sound source localization has been shown to rely on distinct structures. Notably, the caudal subdivisions of auditory cortex and adjacent superior temporal polysensory areas have direct projections to the primary visual cortex. This architecture is hypothesized to enhance stimulus localization in peripheral spaces [72,96]. Such distinctions underline the complexity and specificity of multisensory processing across different contexts. Our study, by detailing the neural correlates of social cue integration, not only enriches the understanding of multisensory processing but also opens avenues for exploring how diverse forms of audiovisual integration come together to influence our overall sensory perception."

b. Results, L104. An intriguing observation is that the LGN is recruited by face videos, but not scrambled faces. Given that this nucleus is usually regarded as a low-level representation where cells analyse small parts of the visual field, this deserves some discussion. Perhaps feedback connections from the cortex could cause this effect?

We acknowledge the reviewer's observation concerning the LGN activation only in response to face videos. As suggested, we added some discussion about that in the modified manuscript, on page 26, which reads:

"Subcortically, face videos preferentially recruited the pulvinar, amygdala, and LGN, in contrast to scrambled faces which did not elicit the same response. This selective activation of the LGN by face videos, and not by scrambled faces, may be influenced by cortical feedback mechanisms. The LGN is typically seen as a primary visual relay, but it is also subject to extensive feedback from cortical areas. This top-down influence from higher-order visual areas to the LGN is known to modulate sensory processing, potentially enhancing or suppressing neural responses based on the context and recognizability of stimuli [68,69]. In the case of our study, the coherent, structured nature of face videos could engage these feedback pathways more robustly compared to scrambled faces, which lack meaningful visual content. Such dynamics align with the concept that perception is not a mere bottom-up process but a complex interplay of sensory and cognitive factors[70]. Further research into these feedback mechanisms could elucidate how the brain discerns and prioritizes meaningful visual stimuli over less coherent ones."

c. Results, L116. the Paxinos parcellation of the NIH marmoset brain atlas - perhaps citation of the original work would be appropriate, given that this is where the criteria for area identification are given.

We thank the reviewer for pointing out the importance of citing the original work of Paxinos in relation to the parcellation of the NIH marmoset brain atlas. In accordance with the reviewer's suggestion, we have added the appropriate citation in all sections of the revised manuscript where Paxinos' parcellation is discussed.
The relevant references can be found on pages 10, 12, 19, 21, 24, 42, and 43 of the revised manuscript.

d. Results, L 128-134. The activations in non-auditory areas such as V1, V2, and primary somatosensory cortex are a bit surprising. It would be useful to have some quantification of the level of these activations, compared to "expected" activations in auditory areas. The color scales in the figures are a bit too compressed to give this information accurately, so a table with the sizes of the effects could help the reader evaluate better the relative magnitudes of the activations.

We are grateful for the reviewer's feedback regarding the activations in non-auditory areas such as V1, V2, and the primary somatosensory cortex observed in our study. To address the reviewer's suggestion for more detailed quantification of these activations, we have included a new supplementary table (Supplementary Table 1) in the revised manuscript. This table offers detailed data on the coordinates of peak activations and their corresponding z-values in both non-auditory and auditory areas during the presentation of marmoset vocalizations.

In addition, responding to a similar suggestion from Reviewer 2, we have also incorporated bar graphs in our supplementary materials (Supplementary Figure 2). These graphs present the beta values for various cortical regions of interest (ROIs) under both unisensory and multisensory conditions. They are intended to provide a clear visual representation of the variations in response levels across different conditions, assisting in a more nuanced understanding of the relative magnitudes of activations in these ROIs.

e. L130. In the Paxinos et al. atlas the superior temporal rostral area (STR) is included in the auditory cortex, being regarded as a likely high-order auditory area based on comparison with Pandya's work in the macaque. In addition, previous research in marmoset (de La Mothe et al. 2006; Burman et al. 2011) has argued for the areas just medial to the medial belt to be regarded as parts of an "extended" auditory cortex. This was originally referred to the proisocortical auditory area by the above authors, but has since been subdivided in the Paxinos atlas (IPro, PaIM, PaIL). Some of these areas show activations by the auditory stimuli. Thus, related to point (d) above, I would be interested to know the size of the effect detected there, relative to other areas.

We appreciate the reviewer's detailed insights regarding the superior temporal rostral area (STR) and its classification as part of the auditory cortex, as well as the mention of areas considered as 'extended' auditory cortex (e.g., IPro, PaIM, PaIL) in marmosets. This nuanced understanding of the auditory cortex subdivisions is indeed crucial for interpreting our findings accurately.

In response to the reviewer's interest in the relative size of the effect detected in these areas, and in connection with the previous point raised, we direct attention to the Supplementary Table 1. This table provides detailed information on the peak activation coordinates and their corresponding z-values for the STR, IPro, PaIM, and PaIL areas, alongside other primary and high-order auditory areas, during the presentation of marmoset vocalizations.

Moreover, to facilitate a comprehensive comparison, we have included bar graphs in Supplementary Figure 2, showcasing beta values for these specific ROIs under both unimodal and multisensory conditions. These visual representations allow for an in-depth evaluation of the effect sizes in these extended auditory cortex areas relative to more traditional auditory regions.

f. P138. Was a specific part of area MST was activated? This question comes from the suggestion advanced by Majka et al. 2019, that area MST as defined in the marmoset atlas may include parts of the superior temporal polysnesory area (TPOc). If the activation was specifically located to the rostral part of MST this could be interesting; if not, please ignore this comment.

We appreciate the reviewer's insightful query regarding the specific activation within the MST area in our study. Upon re-examining our results, we found that the activation for both vocalizations and scrambled vocalization maps, relative to the baseline, appears to be predominantly located in the rostral part of MST. This observation aligns with the suggestion made by Majka et al. 2019 regarding the potential inclusion of parts of the superior temporal polysensory area (TPOc) in the marmoset atlas' definition of MST. Our findings might therefore contribute to the ongoing discussion about the functional anatomy of MST, specifically in relation to its rostral portion and its potential involvement in processing complex auditory stimuli.
This observation has been incorporated into the discussion section of our revised manuscript on pages 26-27, where we note:

"Intriguingly, our results show that activations in the MST region, for both vocalizations and scrambled vocalizations, are predominantly localized in its rostral part. This specific localization supports the hypothesis proposed by Majka et al. [72], suggesting that this part of MST might functionally correspond to the caudal subdivision of the superior temporal polysensory area (TPOc), indicating a complex functional anatomy within MST for auditory processing."

g. Regarding area 32: Reser et al. (2009) made the observation that the marmoset rostrotemporal area (RT) has a direct projection to what he considered "a caudal projection of area 10", based on Price's macaque map. From the section illustrated, this is most likely what is now regarded part of area 32. This would provide additional support to the idea that this region has a auditory cognition role.

We thank the reviewer for highlighting the connection between the marmoset rostrotemporal area (RT) and what is now considered part of area 32, as suggested by Reser et al. (2009). Their observation of direct projections from RT to a region akin to a caudal extension of area 10, based on Price's macaque map, indeed aligns with the idea that area 32 may play a role in auditory cognition. This connection could support our findings regarding

the involvement of area 32 in processing auditory stimuli. We now discuss this aspect in our revised manuscript on page 32:

"Reser et al. [88] provide an important anatomical context to these findings. They observed that the marmoset RT area, part of the core auditory network, has direct projections to a region considered part of area 32. This anatomical connection might contribute to the role of area 32 in auditory cognition, as seen in our study. This underscores the complexity of the involvement of this area in processing auditory and audiovisual social cues."

Reviewer #2 (Remarks to the Author):

In this study, the authors investigated multisensory face and voice processing in marmosets using ultra-high field fMRI. By comparing brain response to face stimuli only, voice stimuli only, and multisensory face-voice stimuli, the authors found multisensory stimuli activated additional fronto-parietal networks beyond unimodal face and voice areas. The experiment is well-designed and carefully conducted, yet essential concerns must be addressed before the study can be published. Major and additional points are listed below.

We appreciate the reviewer's thorough assessment of our paper and their valuable feedback. We have carefully considered and addressed each point raised, with detailed responses and corresponding revisions to the manuscript. We are confident that these changes have strengthened the evidence and clarity of our findings, and we hope that our efforts now meet the reviewer's expectations for publication.

Major points:
1. The process of unimodal and multisensory visual and auditory information has been extensively studied in literature, as the authors cited in the manuscript. Therefore, the novelty of the current study could be clearer—moreover, the motivation and significance of using marmosets as subjects need to be laid out.

We thank the reviewer for highlighting the necessity to clarify the novelty of our study and the rationale for using marmosets as our subjects. While the processing of unimodal and multisensory information is indeed well-established, our study provides novel contributions in the following ways:

1. **Novelty of the study:**
    - **This is the first study to apply ultra-high field (9.4T) fMRI to map the neural architecture underlying multisensory integration of facial and vocal cues in marmosets.**
    - **We reveal new aspects of how New-World monkeys integrate complex social signals, enhancing the comparative scope of the research field predominantly focused on humans and Old-World monkeys.**

2. **Rationale for using Marmosets:**

- **Marmosets display unique social structures and communication behaviors not typically found in Old-World primates, thereby addressing a significant gap in our comparative understanding of primate social communication.**
- **Their growing use as a model in neuroscience, due to their genetic tractability and relevance to human social behavior, positions them as a valuable subject for translational research into neurodevelopmental and psychiatric disorders.**

In response to the reviewer's comments, we have revised our manuscript to emphasize these points. The introduction now explicitly states the unique contributions of our study and justifies the use of marmosets due to their complex social and communicative behaviors. The discussion expands on the evolutionary and translational significance of our findings, highlighting marmosets as a crucial comparative model for understanding primate social cognition and its disorders.

The revised sections can be found on pages 4-5 for the Introduction and pages 35-36 for the Discussion, which now read:

Pages 4-5: *"The field of multisensory integration in primate communication has been extensively explored in Old-World primates and humans. However, in New-World monkeys, the common marmoset (Callithrix jacchus) presents distinctive social behaviors, such as cooperative care of offspring and complex vocal communication, which are less common in Old-World species* [4,55,56]*. These unique characteristics of marmosets provide a valuable comparative model that can enhance our understanding of the evolution of neural mechanisms underlying social behaviors and inform the study of human social cognition and disorders* [57–60]*. In this context, the present study aims to explore the neural circuits for social multisensory integration in the common marmoset, an area so far unexplored. Utilizing ultra-high field MRI, we acquired whole-brain fMRI data from six awake marmosets while the animals were presented with videos of conspecific faces with no sound, conspecific vocalizations with no video, videos of conspecific faces with corresponding vocalizations, and scrambled versions of each of these conditions. By mapping these neural networks, we not only fill a significant gap in the literature but also provide insights that may be crucial for understanding the evolution of primate social communication and its implications for human social cognition, offering a potential avenue for translational research into social behavior and its disorders."*

Pages 35-36: *"In summary, our findings reveal a conserved audiovisual multisensory pathway in marmosets, characterized by the activation of face 'patches' along the occipitotemporal axis and vocal 'patches' in the auditory cortex, intricately linked with prefrontal and cingulate areas. These results suggests that during audiovisual social communication, marmosets recruit a more extensive neural network than what is engaged during the processing of visual and auditory signals alone, highlighting the evolutionary importance of integrating facial and vocal cues for interpreting social interactions* [4,55–57,97,98]*. Given the marmosets' rich social behaviors, which parallel human traits such as prosocial behavior, imitation, and cooperative breeding, our study offers a valuable perspective on the evolution of complex social communication processes within primates. It addresses a critical gap in our comparative understanding of primate neurobiology, illuminating both the specialized multisensory processing in this species and the potential evolutionary mechanisms shared across primates in social communication. Therefore, these*

results not only enhance our knowledge of marmoset neurobiology but also underscore the species' potential as a model for translational research. This is particularly pertinent in the study of neurodevelopmental and psychiatric conditions affecting social interaction, where marmosets may provide key insights [60,99,100]*.”*

2. The scrambled conditions are supposed to be control stimuli. However, they need to be adequately analyzed as controls. Specifically, the actual “face patch” and “voice patch” should derive from contrasting intact vs. scrambled conditions. The actual “multisensory face-voice area” should derive from contrasting intact vs. scrambled multisensory stimuli (as the authors did). Then, all comparisons between multisensory and unisensory conditions should be based on these scramble-controlled images. Without proper control of the scrambled conditions, it is unclear if areas respond to simple visual and auditory stimulation or higher-level communication stimuli.

We appreciate the reviewer's comment on the control analysis of scrambled conditions in our study. To address this concern, we have made the following revisions to our manuscript to ensure a rigorous control analysis:

- **We have now included a new figure (Figure 3 in the revised manuscript) that illustrates the group activation maps that contrast intact versus scrambled conditions for marmoset faces, vocalizations, and their combined presentation.**

- **We have updated the results section with several paragraphs detailing the outcomes of these contrasts. These updates are reflected on pages 8-9 and 13-14 of the revised manuscript, which reads:**

Pages 8-9: *“Next, we identified brain regions more active during marmoset face observation by comparing the marmoset face videos condition with the scrambled condition (i.e., marmoset face videos > scrambled marmoset face videos contrast). The group activation maps depicted in Figure 3A revealed significant activations for marmoset faces compared to scrambled faces in a bilateral network comprising regions in the occipital cortex (i.e., V1, V2, V3, V4, V4t), and the temporal cortex (i.e., TEO, FST, TE1, TE2, TE3, 35, 36, entorhinal cortex). We also observed higher activations in area 19DI on the left hemisphere and in subcortical areas in the right pulvinar and the right amygdala (Figure 3A).*
For detailed quantitative comparisons of activation levels in various cortical regions under these conditions, refer to the bar graphs presented in Supplementary Figure 2. These graphs visually demonstrate the variations in response levels across conditions and provide the statistical insights into the differences between them.”

Pages 13-14: *“To directly identify cortical and subcortical clusters that were more active for vocalizations, we compared the vocal to the scrambled conditions (i.e., marmoset vocalization > scrambled marmoset vocalization contrast). The group map in Figure 3B shows stronger activations for vocalizations in R and RT areas of the core auditory cortex, in AL, ML, RTM, RTL areas of the belt auditory cortex and in RPB and CPM areas of the parabelt auditory cortex. We also found higher activations in occipito-temporal cortex in V1, V2, V2, V4, V4t, V5, TEO, FST, Pga-IPa, TPO, TE3, TE2, TE1, STR, 36, 35, Ent, and PaIM areas. More anteriorly, we found greater activations in the premotor cortex in bilateral area 6 ventral part (6Va) and in right areas*

8 caudal part (8C) and 6DR; in the frontal cortex in bilateral areas 8Av, 45 and 47 medial part (47M) as well as in the right orbitofrontal cortex in areas 47O, 13 lateral (13L), 11 and orbital periallocortex (OPAI). Finally, higher activations were found in bilateral rostral cingulate areas 25, 32, and 29d. At the subcortical level, vocalizations induced stronger activations in the superior colliculus (SC), medial geniculate nucleus (MGN), caudate, pulvinar, and amygdala (Figure 3B)."

- **We have conducted a new conjunction analysis to differentiate common and distinct brain region responses to intact versus scrambled stimuli for marmoset faces, vocalizations, and their combined presentation. This analysis is depicted in the new Figure 5 and described in the corresponding results section on pages 19-20, which reads:**

"We subsequently conducted a conjunction analysis to compare intact versus scrambled stimuli for marmoset faces, vocalizations, and their combined presentation. This was done to discern the common and distinct brain regions involved in processing coherent social signals from faces, vocalizations, and their integrated form. As illustrated in Figure 5, the green and blue regions demonstrate preferential activations for intact vocalizations and faces, respectively. The green regions encompassed the auditory cortex, including core, belt, and parabelt areas, and some motor and somatosensory areas, underscoring vocalization-specific processing. In contrast, the blue regions, localized to sections of temporal areas TE2 and TE1, signal face-specific responses. The red regions, predominantly located in the posterior parietal, prefrontal, and orbitofrontal cortices, highlight areas that are uniquely responsive to a coherent combination of marmoset faces and vocalizations, as opposed to their scrambled versions.
The overlaid regions, delineated in purple, clear blue, yellow, and white, signify converging neural responses between the different contrasts. Specifically, the white regions along the occipitotemporal axis, encompassing V2, V3, V4, V4t, TEO, and parts of FST, TE3, TE2, and TE1, denote a common response to all three intact versus scrambled stimulus categories.
The yellow regions, involving portions of prefrontal area 45, premotor area 6Va, FST, Pga-IPA, MT, STR, PaIM, and early visual areas V1 and V2, show a shared neural response for processing both vocalizations and the integration of faces with corresponding vocalizations. In line with our previous results (Figure 4A), the anterior section of area 32 shows a distinct response to intact vocalizations (in green), the posterior section to the integrated face-vocalization stimuli (in red), and an intermediate zone to both (in yellow). Notably, no regions showed a dual response to both marmoset faces and vocalizations or to marmoset faces and the combination of faces and vocalizations (indicated by the absence of light blue and purple).

In summary, our findings from the conjunction analysis reveal a nuanced neural representation of social stimuli, distinguishing specific and shared processing networks for marmoset faces, vocalizations, and their integrated combinations. A consistent network across the occipitotemporal axis responds to all three modalities when comparing intact to scrambled versions, suggesting an ability to process complex social cues. This network, with the engagement of additional parietal and frontal regions, which are specifically recruited for processing the integrated presentation of marmoset faces with vocalizations, allow multisensory integration in the marmoset brain."

- **We have expanded the discussion to further interpret the significance of the findings on pages 25-27 and pages 32-33, as follow:**

Pages 25-27: "*Face and Vocalization Processing: Face-patch and Voice-patch systems*
The ability to recognize faces is paramount in deciphering the intentions of others, making the differentiation and interpretation of facial expressions vital for social communication [65–67]. Previous fMRI research on face processing in human and nonhuman primates has identified 'face patches' located across temporal and prefrontal cortices, which responded strongly to faces compared to non-face objects or scrambled faces [6–8,10,11,16–19]. Our results from visual stimulation depicting marmoset face videos, as well as comparisons of these videos to their scrambled versions, are consistent with recent investigations into face processing in marmosets. These results emphasize the role of occipito-temporal regions in processing faces and facial expressions [16–18]. These areas display robust activation in areas V2/V3, V4/TEO, V4t/FST, TE2-TE3, corresponding to the previously identified face-patches in marmosets (i.e., patches O (occipital), PV (posterior ventral), PD (posterior dorsal), MD (middle dorsal) and AD (anterior dorsal) respectively) [16,18]. Subcortically, face videos preferentially recruited the pulvinar, amygdala, and LGN, in contrast to scrambled faces which did not elicit the same response. This selective activation of the LGN by face videos, and not by scrambled faces, may be influenced by cortical feedback mechanisms. The LGN is typically seen as a primary visual relay, but it is also subject to extensive feedback from cortical areas. This top-down influence from higher-order visual areas to the LGN is known to modulate sensory processing, potentially enhancing or suppressing neural responses based on the context and recognizability of stimuli [68,69]. In the case of our study, the coherent, structured nature of face videos could engage these feedback pathways more robustly compared to scrambled faces, which lack meaningful visual content. Such dynamics align with the concept that perception is not a mere bottom-up process but a complex interplay of sensory and cognitive factors [70]. Further research into these feedback mechanisms could elucidate how the brain discerns and prioritizes meaningful visual stimuli over less coherent ones.

Research in primate vocalization processing, another fundamental mode of communication in primates, has revealed the existence of selective 'voice patches' in the temporal cortex, specifically along the superior temporal sulcus (STS), and in certain regions of the frontal cortex in humans, macaques, and marmosets [29–32,36–39,42,71]. Our results align with these findings and our recent investigation in marmosets [42], showing similar activations in the primary auditory cortex, rostral cingulate, frontal, and temporal cortices in response to auditory stimulation playing vocalizations. Moreover, we observed comparable activations in the primary auditory cortex and MST for scrambled vocalizations. Intriguingly, our results show that activations in the MST region, for both vocalizations and scrambled vocalizations, are predominantly localized in its rostral part. This specific localization supports the hypothesis proposed by Majka et al. [72], suggesting that this part of MST might functionally correspond to the caudal subdivision of the superior temporal polysensory area (TPOc), indicating a complex functional anatomy within MST for auditory processing.
Our analysis comparing vocalizations with their scrambled vocalizations has revealed additional activations in regions not observed in our initial study [42], including areas in occipital, inferior and lateral temporal, as well as prefrontal cortices. This pattern could be attributed to a notable decrease in activity within the occipitotemporal regions during both conditions, with a more significant deactivation during the presentation of the scrambled conditions (refer to

Supplementary Figure 3 for visualization). Such deactivation was absent in our earlier auditory-only study [42], suggesting that our current experimental design—incorporating visual and audiovisual stimuli either preceding or following the vocalization condition—may influence the neural processing of auditory signals."

Pages 32-33: *"While similarities exist in the neural networks processing multisensory stimuli between intact and scrambled conditions, the activations elicited by intact audiovisual stimuli were more pronounced and extensive, particularly in the prefrontal cortex, compared to their scrambled counterparts. This difference was less marked in the parietal cortex. Directly comparing the two conditions, we observed that intact audiovisual stimuli elicited greater activations across the occipito-temporal axis, within the prefrontal cortex, and notably in the rostral cingulate area 32. Subcortically, the superior colliculus, pulvinar and amygdala were also more activated in response to intact audiovisual condition. The distinct activation patterns within area 32, particularly the differential responses between its posterior and anterior portions, suggest a specialized role in integrating coherent and congruent multisensory information. Specifically, the posterior portion of area 32, which exclusively responds to multisensory stimulation, appears to play a key role in processing complex sensory information, aiding in distinguishing meaningful audiovisual social interactions from nonsensical or mismatched stimuli. This aligns with findings in humans where the anterior ACC is implicated in processing context-dependent multimodal events [89,90]. For instance, Laurienti et al. [54] observed increased activity in the anterior cingulate gyrus and adjacent medial prefrontal cortex when auditory and visual stimuli were contextually congruent, as opposed to when they were mismatched. These observations highlight the advanced cognitive function of area 32 in marmosets, extending beyond basic sensory integration to encompass context and relevance in social communication. Such evidence suggests that the rostral anterior cingulate area 32 might serve as an integration center for context-dependent audiovisual information in social cognition, significantly influencing social interactions.*
Furthermore, the differential activation observed across temporal, prefrontal, and cingulate regions in response to intact versus scrambled conditions could reflect a sophisticated neural mechanism. This mechanism is capable of differentiating relevant audiovisual social information from nonsensical or mismatched stimuli, thereby enhancing the precision and efficacy of primate social communication."

- **The methods section has been updated to include a detailed description of the statistical analyses performed for the contrasts, as outlined on page 42, which reads:**

"Finally, to identify brain regions more activated by intact compared to scrambled stimuli, we contrasted the activations for marmoset face videos, vocalizations and marmoset face videos with corresponding vocalizations conditions against their scrambled versions (i.e., marmoset face videos > scrambled marmoset face videos, vocalizations > scrambled vocalizations, marmoset face videos with vocalizations > scrambled marmoset face videos with scrambled vocalizations). As previously, we also conducted a conjunction map between these three contrasts to observe specific and shared activations between the conditions."

3. The stimuli used in the experiment involve negative facial expressions, and negative cues tend to elicit more robust responses (as the authors discussed in the Introduction). The authors should discuss the results from this perspective.

Following the reviewer's suggestion, we have expanded our discussion to consider the implications of using these specific stimuli. This addition can be found on page 35 of the revised manuscript, where we address the potential impact of negative expressions on neural response patterns. The paragraph reads as follows:

Page 35: *"Furthermore, it is pertinent to address the nature of the stimuli used in our study, specifically the inclusion of negative facial expressions. Previous literature indicates that negative expressions tend to elicit more robust neural responses compared to neutral expressions in temporal, prefrontal, and subcortical areas in both humans and macaques, a pattern also observed in marmosets [6,18,20–25]. Therefore, our findings focused on negative expressions may not fully represent the neural processing associated with other types of facial expressions. Future studies exploring a broader range of emotional expressions in marmosets are necessary to gain a comprehensive understanding of emotional processing in a multisensory social context in this species."*

Other points:
1. In fMRI analysis, the authors first estimated the timing and amplitude of each condition and then used the estimated HRF as the regressor. What is the rationale for this approach？ And is it circular? Why not just use the standard HRF, or model-free methods?

We appreciate the insightful comments of the reviewer and the opportunity to clarify our methodology for fMRI analysis. We have now revised the relevant section of our Methods to more accurately reflect the analysis performed.

We would like to clarify that our use of the BLOCK function in AFNI's 3dDeconvolve to model the HRF is based on the standard shape and timing of the hemodynamic response typical in block design fMRI studies. This widely accepted method assumes a canonical HRF shape, suitable for our study's stimulus duration of 12 seconds.

We have not derived the HRF from our data; instead, we applied this standard BLOCK function as a fixed shape to generate regressors for the GLM analysis. This strategy allows for a consistent evaluation of the BOLD signal in relation to our task conditions and aligns with established fMRI analysis protocols.

The updated Methods section on page 41 now reads:

"We employed the BLOCK function within AFNI's 3dDeconvolve tool to model the hemodynamic response for each condition. This approach uses a predefined canonical HRF shape, parameterized to match our experimental design with 12-seconds stimulus blocks. These parameterized HRFs were used as regressors in a general linear model (GLM), complemented by polynomial detrending and motion parameters. This yielded six T-value maps per run for each subject, corresponding to our experimental conditions."

2. The influence of the multisensory process on unimodal areas would be interesting to investigate. E.g., would multisensory stimuli simply activate more areas beyond unimodal patches or activate more areas AND increase the activity in unimodal areas?

3. Relatedly, adding bar graphs showing the response level of different ROIs under different conditions would help demonstrate the superadditive effect (and when areas do not show a superadditive effect, how their responses vary across conditions).

We thank the reviewer for their insightful suggestions. To address points 2 and 3, we have included additional analyses in the supplementary information of our manuscript. This includes bar graphs showing beta values for various cortical regions of interest (ROIs) under both unisensory and multisensory conditions, presented as Supplementary Figure 2. These graphs demonstrate response level variations across and between different conditions and highlight the superadditive effect in these ROIs.

Additionally, we have added references to this figure in the revised manuscript's results section, specifically on pages 8, 9, 12, and 15. Furthermore, on page 23, we included a new paragraph detailing the statistical analysis performed to demonstrate the superadditive effect. This analysis critically examines whether multisensory stimuli activate more areas beyond unimodal patches and also increase activity within these unimodal areas.

The relevant sections from the revised manuscript are as follows:

Pages 8-9: *"For detailed quantitative comparisons of activation levels in various cortical regions under these conditions, refer to the bar graphs presented in Supplementary Figure 2. These graphs visually demonstrate the variations in response levels across conditions and provide the statistical insights into the differences between them."*

Page 12: *"For quantitative comparisons of activation levels in various cortical regions under these auditory conditions, refer to the bar graphs presented in Supplementary Figure 2."*

Page 15: *"Quantitative comparisons of activation levels in these cortical regions are further detailed in Supplementary Figure 2."*

Page 23: *"To provide a more detailed understanding of these observations, we included bar graphs in the supplementary information (Supplementary Figure 2), which display the beta values of various regions of interest (ROIs) under the different conditions. Additionally, our statistical analysis, which compared the beta values in the multisensory condition (faces with vocalizations) against the combined beta values from the unimodal conditions (faces + vocalizations) across these ROIs, revealed significant superadditive effects in all the ROIs, except for the frontal region 11 of the left hemisphere (all p-values < 0.01 except for area 11 left hemisphere). This analysis underscores the nuanced and robust nature of the superadditive response, especially in regions involved in sensory integration and higher-order processing."*

In the methods section (page 43), we have also elaborated on the process employed to generate the bar graphs. This includes details on the extraction of ROIs and the statistical methods used for analysis.

The method section now reads:
"[...] In order to visualize the response levels under the different conditions, we extracted 68 cortical ROIs from each hemisphere based on the Paxinos parcellation [63] of the NIH marmoset brain atlas [64]. These ROIs were categorized according to their cortical position: 9 ROIs corresponded to visual areas, 7 to ventrolateral prefrontal and orbital frontal areas, 6 to dorsolateral prefrontal and premotor areas, 9 to posterior cingulate and medial prefrontal areas, 8 to posterior parietal areas, 8 to lateral, inferior, and ventral temporal areas, 13 to auditory areas, and 8 to the insula and other regions in the lateral sulcus. Beta values for each condition and run were extracted from the resultant regression coefficient maps using AFNI's 3dmaskave function in each ROIs. Differences between conditions were computed using two-sided paired t-tests with FDR post-hoc correction ($p < .05$) using a custom-written Matlab script (R022a, The Mathworks). This analytical approach enabled us to conduct a detailed examination of response levels in specific cortical areas under the different auditory, visual, and audiovisual conditions. Additionally, within each ROIs, we computed the difference between the multisensory condition and the sum of the unisensory visual and auditory conditions to investigate the superadditive effect using two-sided paired t-tests ($p < .05$)."

4. Only the left hemisphere is shown in Figure 1, whereas the rest shows both hemispheres. It would be good to be consistent.

We thank the reviewer for pointing out the inconsistency in the representation of hemispheres in our figures. To address this and provide a consistent and comprehensive view of our results, we have created additional supplementary figures that specifically visualize the activation maps of the right hemisphere.

These new supplementary figures (Supplementary Figures 1A, 1B, 1C, 1D, 1E, and 1F) present detailed visualizations of the right hemisphere corresponding to the various conditions examined in our study. We have also inserted sentences in the revised manuscript's results section to guide readers to these supplementary figures. The references to these supplementary figures are as follows:

Page 8: *"The group activation maps for each condition, focusing on the left hemisphere, are depicted in Figures 2A and 2D. For a detailed visualization of the activation maps of the right hemisphere, see Supplementary Figure 1A et 1D."*

Page 12: *"The group activation maps for these conditions, focusing on the left hemisphere, are depicted in Figures 2B and 2E. For detailed visualization of the activation maps of the right hemisphere under these conditions, see Supplementary Figures 1B and 1E."*

Page 14: *"In the intact audiovisual condition, as illustrated in Fig. 2C (see Supplementary Figure 1C for visualization of the right hemisphere) […] In the scrambled audiovisual condition depicted in Figure 2F (see Supplementary Figure 1F for visualization of the right hemisphere)"*

Reviewer #3 (Remarks to the Author):

This study investigates the integration of auditory and visual vocalizations in the marmoset brain using fMRI at 9.4T. By comparing brain-wide responses to face movies alone, acoustic vocalizations alone, and their combination, the authors reveal a broad and apparently robust network of cortical and subcortical areas whose activation enhanced by, or exclusive to, multimodal stimulation. The authors link their findings in the marmoset to those in humans and macaques, highlighting prefrontal cortical areas that may be involved in complex social perception.

I really like this paper, as the methods are impressive, the design is straightforward, the results are described succinctly, and the findings are striking. There is little to criticize. I list a few points below that the authors may want to take into consideration.

We appreciate the very positive and constructive feedback provided by the reviewer on our manuscript. It is indeed gratifying to learn that the methods, design, and findings of our study have been well-received and positively resonated with the reviewer.

In response to the insightful points raised for further enhancement of our manuscript, we have undertaken thorough revisions and have incorporated changes accordingly. Below, we address each point raised by the reviewer, highlighted in bold, detailing the specific amendments made to our manuscript.

Points to consider:

The authors jump right into the results in Figure 1, perhaps because they have previously described their fMRI methods extensively in other papers. I did, however, find it somewhat wanting for a bit more description about the block design, fMRI brain coverage, analysis assumptions etc. Some readers at this point in the paper might look at the Methods to discover Figure 5, but I did not. If it were me, I would spend a figure on the methods, including some aspects of the imaging at 9.4T, which I think is a strength of this study, rather than a liability. Also, I would show off the fact that there are 6 subjects, novel methods, etc in supplemental material. I don't think any of this will lead to confusion that this is a methods paper. Just a suggestion.

We appreciate the reviewer's constructive feedback, which has highlighted the significance of providing a clear and comprehensive description of our fMRI methodology within the main text. In accordance with the suggestions:

- **We have updated what was previously Figure 5 to become Figure 1 in the revised manuscript. This updated figure now includes detailed visual information about the 9.4T imaging capabilities, such as gradient strength and the multi-channel receive**

coil, along with the temporal signal-to-noise ratio (tSNR) that can be achieved with our setup. Acting as a visual abstract, it provides immediate insight into the key methodological aspects of our study within the Results section, ensuring readers have a clear understanding of the experimental setup.

- **A new paragraph has been introduced at the beginning of the Results section in the revised manuscript. This paragraph concisely outlines the methods used in our study, providing readers with a clear understanding of the experimental context and design before delving into the detailed findings Additionally, it includes a reference to our recent protocol paper, offering readers further insight into the in-depth methodological aspects of our research.**
- **Although we have not created a separate supplemental materials section, we believe that the citation of our recently published protocol paper (Zanini, A., Dureux, A., et al., 2023. "In vivo functional brain mapping using ultra-high-field fMRI in awake common marmosets." STAR protocols) provides comprehensive methodological details pertinent to our study.**

The new introductory methods paragraph in the Results section reads:

Pages 5-6: *"In this study, we employed ultra-high field fMRI at 9.4T to examine the neural correlates of multisensory processing in six awake common marmoset monkeys. Data were collected using a custom-built gradient coil with a 15-cm inner diameter and a maximum gradient strength of 1.5 mT/m/A, coupled with an 8-channel receive coil [61] (Figure 1A). The image quality of the functional runs was assessed by the temporal signal-to-noise ratio (tSNR), demonstrating high-quality data acquisition at this field strength (Figure 1B).*
During scanning sessions, monkeys were positioned in a sphinx posture within an MRI-compatible restraint system, with their heads were secured using a head post and MRI-compatible auditory tubes placed directly into their ear canals.
We acquired eight functional runs per animal, covering the whole brain with 42 axial slices at an isotropic resolution of 0.5 mm (Figure 1C). To mitigate the masking of auditory stimuli by scanner noise, we implemented a continuous acquisition paradigm incorporating silent periods of 1.5 seconds within each 3-second TR [42]. Our block-design experiment utilized various stimuli, including marmoset face videos, vocalizations, marmoset face videos with corresponding vocalizations, and their scrambled versions, each presented for 12 sec in a randomized sequence interspersed with 15-second baseline periods (Figures 1D and 1E).
For detailed information on all methodological specifics, including surgical and anesthesia procedures, MRI training, positioning of marmosets within the MRI-compatible body restraint, fMRI scanning at 9.4T, and preprocessing and statistical analysis of functional images, please refer to the methods section and our group's recent protocol paper [62]."

The authors mention area 32 quite a bit, pointing to its role in processing coherent stimuli. That's interesting, but there's not much to grab onto there and I found the discussion essentially a restatement of the results.

A couple of other areas might also deserve mention. For example, is it known that the premotor areas respond exclusively to auditory stimuli (Fig 3A, showing this is only true for intact acoustic vocalizations)? Might that be related, for example, to directing the orientation toward a vocalizing

conspecific (before the face is visualized? Also, the very strong multimodal enhancement of subcortical structures (SC, Pul), and peripheral visual cortex (e.g. calcarine V1) was striking. Somehow the combination of cues leads the brain to resonate with activity in a way that may not fit cleanly into how people traditionally think about "multimodal integration". The authors might want to make a bigger deal about this. It's not surprising that, as they say on p.12, "multisensory processing is not a simple summation of each modality but also involves additional brain regions". But this result seems like much more significant for understanding the integration of social sensory information than a refutation of that standard null hypotheses about additivity.

We appreciate the reviewer's comments and recommendations regarding our manuscript, particularly concerning modifications in the discussion.

In response to these suggestions, we have carefully revised the Discussion section of our manuscript. We have expanded our discussion on area 32 and we have included additional paragraphs regarding the specific activation of premotor areas in response to auditory stimuli, and the multimodal enhancement observed in our findings. These revisions can be found on pages 31, 32, 33 and 34 of the revised manuscript, which reads:

Page 31: *"Moreover, we observed specific activations in parts of the premotor regions (e.g., 6DC) and the primary motor cortex in response to intact marmoset vocalizations. These activations may reflect a preparatory response for orienting toward a vocalizing conspecific, which is essential for primate social interactions. This aligns with findings from Roy et al. [87], who demonstrated the premotor cortex's involvement both before and during self-initiated vocalizations when marmosets engaged in vocal exchanges with conspecifics. Notably, a subset of premotor cortex neurons was activated specifically by vocal production and not by other orofacial movements, such as licking. This suggests that the premotor cortex may either control specific muscles involved in vocal production, such as those in the larynx, or represent upstream control signals for initiating vocalization [87]. Our fMRI findings suggest an anticipatory neural mechanism in marmosets, potentially oriented towards the source of a vocalizing conspecifics. Such a mechanism could be crucial for appropriate social responses in a context-dependent manner."*

Pages 31-33: *"It is important to note that, although not previously reported in macaques and humans, we show that the rostral cingulate area 32 participates in the integration of visual and auditory stimuli. Interestingly, only the posterior portion of this areas responded exclusively to multisensory stimulation. In contrast, the anterior portion activated solely in response to auditory cues. The intervening region displayed activation in both auditory and combined audiovisual conditions. Nevertheless, these findings held true only for intact, coherent stimuli and not for the scrambled, incoherent versions. Reser et al. [88] provide an important anatomical context to these findings. They observed that the marmoset RT area, part of the core auditory network, has direct projections to a region considered part of area 32. This anatomical connection might contribute to the role of area 32 in auditory cognition, as seen in our study. This underscores the complexity of the involvement of this area in processing auditory and audiovisual social cues.*

While similarities exist in the neural networks processing multisensory stimuli between intact and scrambled conditions, the activations elicited by intact audiovisual stimuli were more pronounced and extensive, particularly in the prefrontal cortex, compared to their scrambled counterparts. This difference was less marked in the parietal cortex. Directly comparing the two

conditions, we observed that intact audiovisual stimuli elicited greater activations across the occipito-temporal axis, within the prefrontal cortex, and notably in the rostral cingulate area 32. Subcortically, the superior colliculus, pulvinar and amygdala were also more activated in response to intact audiovisual condition. The distinct activation patterns within area 32, particularly the differential responses between its posterior and anterior portions, suggest a specialized role in integrating coherent and congruent multisensory information. Specifically, the posterior portion of area 32, which exclusively responds to multisensory stimulation, appears to play a key role in processing complex sensory information, aiding in distinguishing meaningful audiovisual social interactions from nonsensical or mismatched stimuli. This aligns with findings in humans where the anterior ACC is implicated in processing context-dependent multimodal events [89,90]. For instance, Laurienti et al. [54] observed increased activity in the anterior cingulate gyrus and adjacent medial prefrontal cortex when auditory and visual stimuli were contextually congruent, as opposed to when they were mismatched. These observations highlight the advanced cognitive function of area 32 in marmosets, extending beyond basic sensory integration to encompass context and relevance in social communication. Such evidence suggests that the rostral anterior cingulate area 32 might serve as an integration center for context-dependent audiovisual information in social cognition, significantly influencing social interactions.

Furthermore, the differential activation observed across temporal, prefrontal, and cingulate regions in response to intact versus scrambled conditions could reflect a sophisticated neural mechanism. This mechanism is capable of differentiating relevant audiovisual social information from nonsensical or mismatched stimuli, thereby enhancing the precision and efficacy of primate social communication."

Pages 33-34: *"The principle of superadditivity, in which multisensory responses exceed the sum of the linear additive responses to the unimodal stimuli, has been advocated by some researchers as a requirement for brain regions involved in multisensory integration [91,92]. In line with this, we assessed the augmented responses to the audiovisual multimodal conditions in comparison to the sum of visual and auditory unimodal conditions, aiming to identify the regions exhibiting a superadditive effect. Our findings revealed that this augmented response was not merely a simple summation of unimodal stimuli, but rather a complex interplay of activation across temporal, parietal, cingulate, lateral, and medial prefrontal areas. This suggests a synergistic enhancement of sensory processing that goes beyond mere additive effects. Both visual and auditory areas also demonstrated significant differences between multimodal and unimodal stimulations, although the more pronounced differences in activations were situated in the temporal, parietal, and cingulate areas. Subcortically, structures such as the superior colliculus, caudate, MGN, pulvinar, and amygdala exhibited a stronger response to the multisensory condition compared to the sum of unimodal conditions. The superadditive effect, especially pronounced in these areas, contributes to the efficiency and robustness of multisensory integration, enhancing the marmosets' ability to perceive, interpret, and respond to complex social cues.*

These enhancements, transcending traditional models of sensory integration, resonate with the dynamic and context-sensitive neural mechanisms proposed by Ghazanfar and Schroeder [93] and Stein and Stanford [94]. This intricate interplay between sensory modalities, particularly evident in social contexts, suggests a nuanced and perhaps evolutionarily conserved mechanism where the brain integrates complex social cues in a way that significantly enhances overall perceptual experience. This implies that the integration of social sensory information in marmosets, and potentially other primates, relies on sophisticated neural networks extending beyond mere sensory

combination, playing a crucial role in the rapid and effective processing of social cues during communication [93,94]*.''*

REVIEWERS' COMMENTS:

Reviewer #1 (Remarks to the Author):

The authors have revised the manuscript according to each of my suggestions. As I mentioned earlier it was already quite polished, but I like it even better now. The new bar graphs and table in the supplementary material are very useful. Congratulations on outstanding work.

Reviewer #2 (Remarks to the Author):

The authors have properly addressed my previous comments. I appreciate it and do not have further concerns.

Reviewer #3 (Remarks to the Author):

The authors have carefully considered and responded to my comments, and I am happy to recommend publication.
